# Field plants strategically regulate water uptake from different soil depths by spatiotemporally adjusting their radial root hydraulic conductivity

**DOI:** 10.1111/nph.70013

**Published:** 2025-03-19

**Authors:** William Rickard, Imrul Hossain, Xiaoxian Zhang, Hannah V. Cooper, Sacha J. Mooney, Malcolm J. Hawkesford, W. Richard Whalley

**Affiliations:** ^1^ Sustainable Crops and Soils, Rothamsted Research, West Common Harpenden AL5 2JQ UK; ^2^ School of Biosciences University of Nottingham, Sutton Bonington Campus Loughborough, Leicestershire LE12 5RD UK

**Keywords:** *in situ* measurements, permanent grass, radial root water permeability, root water potential, root water uptake, winter wheat

## Abstract

- Plants modify their root hydraulics to maintain water status and strategically use soil water, but how they achieve this in the field conditions remains elusive.
- We developed a method to measure and calculate daily root water uptake, root water potential, and radial root water permeability at different depths in a wheat (*Triticum aestivum* L.) field and a permanent grassland dominated by ryegrass (*Lolium perenne* L.).
- During the drying processes, both plant systems reduced the radial water permeability of their shallow roots to limit topsoil water uptake, while increasing the radial water permeability of their roots in the subsoil to enhance water extraction. Conversely, after the topsoil was rewetted, both plant systems increased the radial water permeability of their shallow roots to enhance water extraction, while reducing the radial water permeability of their roots in the subsoil to limit water uptake.
- Root water uptake in the subsoil was more influenced by the topsoil water than by the subsoil water. The topsoil water serves both as a resource and a signal, coordinating optimal water uptake from different soil depths. These findings have important implications for understanding how plants cope with periodic water stress in the field and for screening drought‐tolerant crop varieties.

## Abstract 2

See also the Commentary on this article by Burks & Tumber‐Dávila, **247**: 436–438

## Introduction

With drought occurrences projected to increase due to climate change, breeding crops tolerant to water stress has become crucial to sustaining crop yields and meeting the growing demand for food (Davies & Bennett, 2015). Among various techniques, developing cultivars with deep roots and improved rhizosphere has been proposed as a potential solution to address this challenge (Lynch, 2013, 2019; Gao *et al*., 2016; Rabbi *et al*., 2018; Hallett *et al*., 2022). However, root water uptake depends not only on root architecture and its rhizosphere (Zhu *et al*., 2024), but also on other abiotic and biotic factors (Vadez, 2014; Q. Sun *et al*., 2021). Phenotyping root morphology and analysing the rhizosphere alone is thus insufficient to determine the water use efficiency of plants, and understanding the response of other root traits to environmental changes is also important (Vadez, 2014). In fact, experimental observations have shown that not all plants with deep roots increased their water uptake from the deep soil when the topsoil dried (Prechsl *et al*., 2015; Rasmussen *et al*., 2020; Gessler *et al*., 2022; Deseano Diaz *et al*., 2023), and a recent meta‐analysis showed that root depth does not necessarily equate to root water uptake depth (Bachofen *et al*., 2024). These suggest the existence of additional mechanisms that regulate root water uptake from different soil layers (Kulmatiski & Beard, 2013).

Water ascent in plants is driven by a water potential gradient between soil and leaves. Plants regulate this process by modifying their hydraulic conductance in different organs (Bartlett *et al*., 2016). In the aboveground, plants cope with water stress by stomatal closure (Hopmans & Bristow, 2002; Carminati & Javaux, 2020; Corso *et al*., 2020), and xylem embolisation (Loepfe *et al*., 2007; Bartlett *et al*., 2016; Scoffoni *et al*., 2017; Gao *et al*., 2020), while the strategies plants use to extract water from different soil layers in the field remain elusive (Kühnhammer *et al*., 2020). Root water uptake involves two distinct yet interconnected processes: radial water flow from the rhizosphere into root xylem vessels, and axial water flow through the xylem vessels (Vadez, 2014). Compared to axial water flow, the pathways through which water moves from the rhizosphere into the xylem are multiple and complicated (Steudle & Peterson, 1998; Johnson *et al*., 2014; Domec *et al*., 2021). Recent research indicated that the resistances of these pathways control not only water flow in the soil–plant–atmosphere system but also stomatal closure when the soil dried (Carminati & Javaux, 2020; Abdalla *et al*., 2021; Cai *et al*., 2022; Yang *et al*., 2023).

The molecular and biophysical mechanisms regulating the response of radial root hydraulic conductivity to water stress are fairly understood for a single root segment (Maurel & Nacry, 2020). The difficulty is in extrapolating these findings to the field where soil water varies spatiotemporally (Tardieu *et al*., 1992). Unlike controlled pots and hydroponic experiments that intentionally dehydrated part of a root system and kept the other part adequately hydrated for a limited period (Zhang & Davies, 1987; Dodd *et al*., 2010; Kreszies *et al*., 2020; Suresh *et al*., 2024), roots at different depths in the field represent different parts or branches of the same root system, where the shallow roots experience periodic wetting–drying cycles due to irregular precipitation and irrigation, while the deep roots generally stay in a relatively stable and moist condition. It has been found that roots in the subsoil could increase their water uptake as a compensation when the topsoil dried, indicating the presence of signals that coordinate root water uptake from different soil depths (Simunek & Hopmans, 2009; Couvreur *et al*., 2012; Thomas *et al*., 2020). Theoretical modelling indicates plants can increase subsoil water uptake by either decreasing (more negative) its root water potential or increasing the ratio of the axial root conductance to the radial root hydraulic conductance (Draye *et al*., 2010). However, experimental studies on compensatory root water uptake have produced mixed results, with some finding compensatory uptake (Johnson *et al*., 2014; Thomas *et al*., 2020; Müllers *et al*., 2023), while others showed no or limited increase in subsoil water uptake when shallow roots experienced water stress (Gessler *et al*., 2022; Müllers *et al*., 2023).

Plants under water stress tend to maintain their water status by modifying their root hydraulic network to regulate water uptake (Clarkson *et al*., 2000; Maurel *et al*., 2010). For example, column experiments have shown that in the absence of water stress, shallow roots of some plants were more effective in taking up water than their roots in the subsoil (Müllers *et al*., 2022), while under water stress, the plants reduced the hydraulic conductance of their shallow roots, accompanied by an increase in hydraulic conductivity of their roots in the subsoil to sustain transpiration (Müllers *et al*., 2023). Most experimental studies on root response to water stress have focused on changes in root hydraulic conductance of plants grown in pots or hydroponic systems by imposing a water stress for a limited period (Hu *et al*., 2011; Müllers *et al*., 2023). In the field, plants experience periodic water stress, and their roots penetrate much deeper. The strategies plants use to cope with such periodic water stress in the field are poorly understood because of the difficulties associated with *in situ* measurements. This paper aims to bridge this knowledge gap.

We developed a method to continuously measure and calculate daily root water uptake, root water potential, and radial root water permeability at different depths in a wheat (*Triticum aestivum* L.) field and a permanent grassland dominated by perennial ryegrass (*Lolium perenne* L.) from 1 April to 30 June 2022. During this period, there were two significant rainfall events. These allow us to elucidate the strategies the two plant systems used to cope with periodic water stress and the differences in their use of these strategies.

## Materials and Methods

### Experimental site and soil water measurement

The field experiment was conducted at the long‐term ley‐arable experiment at Highfield in Rothamsted Research (51°48′10.0″N, 0°21′57.7″W). Details of the experiment are available online (https://www.era.rothamsted.ac.uk/experiment/rrn1). In brief, the experiment consists of 47 6 m × 50 m plots managed as permanent grassland, bare fallow, and arable. The experiment began in 1948, but the permanent grassland dated back at least to 1838. The arable plots with continuous wheat (*Triticum aestivum* L.) started in 1948 and have been ploughed regularly with a mouldboard to a depth of 23 cm. The permanent grass is mown once a year, with the hay removed. The wheat is fertilised with ammonium nitrate at 220 kg‐N ha^−1^. The soil is a silty clay loam, part of the Batcombe series or a Chromic Luvisol in the FAO soil classification (Gregory *et al*., 2016). The average soil bulk density in the arable and the grassland plots is 1.37 and 1.21, respectively.

A trenching machine (TRX‐16‐THT‐0857) with a width of 30 cm was used to excavate the soil to a depth of 45 cm on 1 November 2021, with the soil evacuated from each layer kept separately. Before trenching, we stripped the grasses and their root systems. Soil moisture sensors (Delta SM150T, Cambridge, UK) were installed by gently pushing their prongs into the wall of the trench at the depths of 15, 30, and 45 cm. After sensor testing, the trench was backfilled with the excavated soil to ensure that bulk density and soil stratification of the backfill were as close as possible to those before the excavation. The backfilled trench in the arable plot was re‐seeded, and that in the grassland was re‐turfed by the stripped grasses. All sensors were connected to a GP2 data logger (Delta T, Cambridge, UK), set to record readings every 15 min.

### Root length distributions and water release curves

Cylindrical soil cores, 100 cm long with an internal diameter of 5 cm, were taken from the two plant systems at the end of June using an auger proximal to where the sensors were installed (Van Walt Ltd, Surrey, UK). There were three replicates for each system. Root length density in different soil depths was measured using the core‐break method by cutting 5 cm from the top of the core first to reveal a fresh face (White & Kirkegaard, 2010; Hodgkinson *et al*., 2017), and then every 10 cm. On each face, the number of roots was counted three times with the core rotated 120° after each counting. The number of roots at each breakpoint is the sum of the roots on both sides of the breakpoint. Root‐count data were converted into root length density, based on the assumption that the roots were parallel with the axis of the cores (White & Kirkegaard, 2010). Intact soil cores were also taken from the same sites to measure water release curves, with matric potential from −0.5 to −30 kPa measured using the standard tension plate and potential from −100 to −1500 kPa measured using the standard pressure plate apparatus. The details and results are given in the Supporting Information (Methods S1; Fig. S1).

### Root water uptake rate

**Fig. 1:**
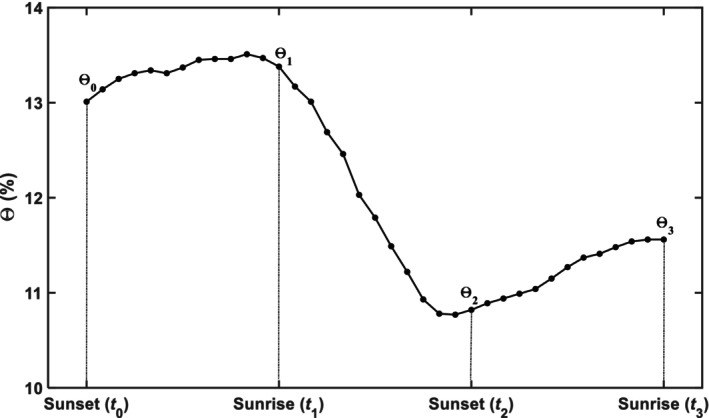
Schematic showing hourly changes in soil water content over a single day and its preceding and following nights.

The change in soil moisture measured by a sensor is the consequence of root uptake and water distribution in the soil profile. It is collectively described by Simunek & Hopmans (2009) and Zhang *et al*. (2020)

(Eqn 1) $$\frac{\partial \Theta }{\partial t}=-\frac{\partial q}{\partial z}-s$$

where Θ is volumetric soil water content, *t* is time (d), *q* is the volume of water flowing across a unit horizontal sectional area (cm d^−1^), *z* is soil depth (cm), and *s* is the volume of water extracted by roots in a unit volume of soil at depth *z* (d^−1^). Fig. 1 shows illustratively the hourly changes in soil moisture content measured continuously in a single day and its preceding and following nights in the wheat field. As root water uptake occurs primarily in the daytime and nighttime transpiration is negligible, integrating Eqn 1 separately over the two nights and the one daytime gives (Li *et al*., 2002)

(Eqn 2) $$\begin{array}{l} \int_{t_{0}}^{t_{1}}\frac{\partial \Theta }{\partial t}dt=\left(\Theta_{1}-\Theta_{0}\right)=-\frac{\partial Q_{1}}{\partial z}\\ \int_{t_{1}}^{t_{2}}\frac{\partial \Theta }{\partial t}dt=\left(\Theta_{2}-\Theta_{1}\right)=-\frac{\partial Q_{2}}{\partial z}-\left(t_{2}-t_{1}\right)s^{\prime }\\ \int_{t_{2}}^{t_{3}}\frac{\partial \Theta }{\partial t}dt=\left(\Theta_{3}-\Theta_{2}\right)=-\frac{\partial Q_{3}}{\partial z}\\ Q_{1}=\int_{t_{0}}^{t_{1}}\mathrm{qdt},\;Q_{2}=\int_{t_{1}}^{t_{2}}\mathrm{qdt},\;Q_{3}=\int_{t_{2}}^{t_{3}}\mathrm{qdt}\\ s^{\prime }=\frac{1}{t_{2}-t_{1}}\int_{t_{1}}^{t_{2}}\mathrm{sdt} \end{array}$$

Approximating $\partial Q_{2}/\partial z$ by

(Eqn 3) $$\frac{\partial Q_{2}}{\partial z}\approx \frac{1}{2}\left(\frac{1}{t_{1}-t_{0}}\frac{\partial Q_{1}}{\partial z}+\frac{1}{t_{3}-t_{2}}\frac{\partial Q_{3}}{\partial z}\right)\left(t_{2}-t_{1}\right)$$

gives the average root water uptake rate between *t*_1_ and *t*_2_ as follows:

(Eqn 4) $$s^{\prime }=\frac{\Theta_{1}-\Theta_{2}}{t_{2}-t_{1}}+\frac{1}{2}\left[\frac{\Theta_{1}-\Theta_{0}}{t_{1}-t_{0}}+\frac{\Theta_{3}-\Theta_{2}}{t_{3}-t_{2}}\right]$$

where the second term on the right‐hand side represents soil moisture change induced by water flow across soil sections.

### Radial root water permeability and root water potential

Root length, radial root water permeability, and water potential difference between the soil–root interface and the xylem vessels in the stele regulate water flow from soil into the root. For a cylindrical root segment with length *L*_i_, root radius *R*_i_, stele radial *r*_i_, and intrinsic water permeability *k*_i_, the relationship between them and water uptake of the root segment is derived in Methods S2. If there are *N* root segments in the soil layer at depth z, root water uptake in the layer is the sum of water uptake of the *N* root segments:

(Eqn 5) $$Q=2\pi \sum_{i=1}^{N}L_{i}k_{i}\frac{\psi -\psi_{i}}{\ln\left(R_{i}/r_{i}\right)}$$

where *ψ* and *ψ*_i_ are soil matric potential and water potential in the i^th^ root segment at depth *z*, respectively. In Eqn 5, *k*_i_ is the ability of the peripheral cell layers (including epidermis, cortex, and endodermis) to transport water from the root–soil surface to the xylem vessels in the stele. As explained in Methods S2, it differs from the radial root hydraulic conductivity and radial root hydraulic conductance, which depend on the water permeability of the peripheral cell layers, root diameter, and stele diameter. As with soil hydraulic conductivity, if water potential is expressed as water head, the unit of *k*_i_ is cm d^−1^, while if water potential is expressed as pressure (MPa), the unit of *k*_i_ is cm^2^ d^−1^ MPa^−1^. At depth *z*, the radial root water permeability varies between root segments (McCormack *et al*., 2015); we rewrite Eqn 5 as follows by introducing a volume‐average radial root water permeability (*k*_r_) and a volume‐average root water potential (*ψ*_0_):

(Eqn 6) $$\begin{array}{l} Q=2\pi k_{r}L\left(\psi -\psi_{0}\right)\\ L=\sum_{i=1}^{N}L_{i}\\ k_{r}=\sum_{i=1}^{N}\frac{L_{i}}{L}\frac{k_{i}}{\ln\left(R_{i}/r_{i}\right)}\\ \psi_{0}=\sum_{i=1}^{N}k_{i}\psi_{i}/k_{r} \end{array}$$

where *L* represents the length of the *N* root segments. Average water uptake per unit root length is $q=Q/L=2\pi k_{r}\left(\psi -\psi_{0}\right)$. Unlike *k*_i_ for an individual root segment, which is the intrinsic property of its peripheral cell layers, the volume average makes the effective radial water permeability (*k*_r_) depend on both water permeability of the peripheral cell layers and the ratio of root radius to stele radius of each individual root segment.

Eqn 6 applies to roots in both the topsoil and subsoil. If root water uptake rate (*q*) increases linearly with soil matric potential (*ψ*), the effective root water potential is the interception of *q* at *ψ*, and the effective radial root water permeability is the slope scaled by 2π. If *q* increases with *ψ* nonlinearly, both *k*_r_ and *ψ*_0_ vary with *ψ*. As *ψ* changed with time in our experiment, *k*_r_ and *ψ*_0_ also varied with time. When time increases incrementally from *t* to *t* + Δ*t*, after a rainfall, for example, soil matric potential at depth *z* decreases from *ψ* to *ψ* + Δ*ψ*, due to root water uptake. If Δ*t* is small and root growth during Δ*t* is negligible and *Q* decreases with *ψ* linearly, the effective root water potential and radial root water permeability can be approximated as constant. From Eqn 6, they can be calculated from

(Eqn 7) $$\begin{array}{l} 2\pi k_{r}L\left(t\right)=\frac{Q\left(\psi +\Delta \psi \right)-Q\left(\psi \right)}{\left(\psi +\Delta \psi -\psi_{0}\right)-\left(\psi -\psi_{0}\right)}=\frac{Q\left(\psi +\Delta \psi \right)-Q\left(\psi \right)}{\Delta \psi }\\ \psi_{0}=\psi +\frac{\Delta \psi }{Q\left(\psi +\Delta \psi \right)-Q\left(\psi \right)}Q\left(\psi \right) \end{array}$$

If root water potential (*ψ*_0_) is known, instead of using Eqn 7, the effective radial root water permeability can be calculated as follows using the measured *Q* and *ψ* as described above:

(Eqn 8) $$k_{r}=\frac{1}{2\mathrm{\pi L}\left(t\right)}\cdot \frac{Q\left(\psi \right)}{\psi -\psi_{0}}$$

We will discuss how Eqns 7 and 8 were used.

Calculating radial root water permeability requires root length *L*. Roots grow in the field, but their growth rates are difficult to measure *in situ*. To ensure accuracy and representativeness, sampling needs to target the roots near the sensors. However, destructively sampling in the proximity of the sensors damages roots and disturbs soil, compromising data accuracy. Conversely, sampling roots in distant sites does not represent roots measured by the sensors because of spatial heterogeneity. A compromise is to measure roots near the sensors at the end of the experiment and then use extrapolations to evaluate how root growth impacts the calculated results. From the onset to the end of the experiment, we approximated root growth by

(Eqn 9) $$L\left(t\right)=\beta \left(t_{m}-t\right)+L_{m}=L_{m}\left[\alpha \left(t_{m}-t\right)+1\right],$$

where *L*(*t*) is root length at time *t*, *β* (cm cm^−3^ d^−1^) is a root‐growth parameter, *L*_m_ is the root length density (cm cm^−3^) measured at time *t*_m_ (the end of the experiment), and $\alpha =\beta /L_{m}$ (d^−1^) is root growth rate, with *α* = 0 representing no root growth.

### Statistics

The changes in root water uptake with soil water content and soil matric potential in each soil layer were fitted to predefined functions, including linear function, power‐law function, and exponential function. Measured by the *P*‐value and the coefficient of determination, the best‐fitting function is the one that yielded the highest *P*‐value. Root water uptake and soil matric potential at the same depth were divided into a before‐rainfall group and an after‐rainfall group, and the data in each group was fitted separately to predefined functions. Significant differences in the fittings between the two groups were calculated using the analysis of covariance (ANCOVA).

## Results

### Root water uptake in different soil layers

**Fig. 2:**
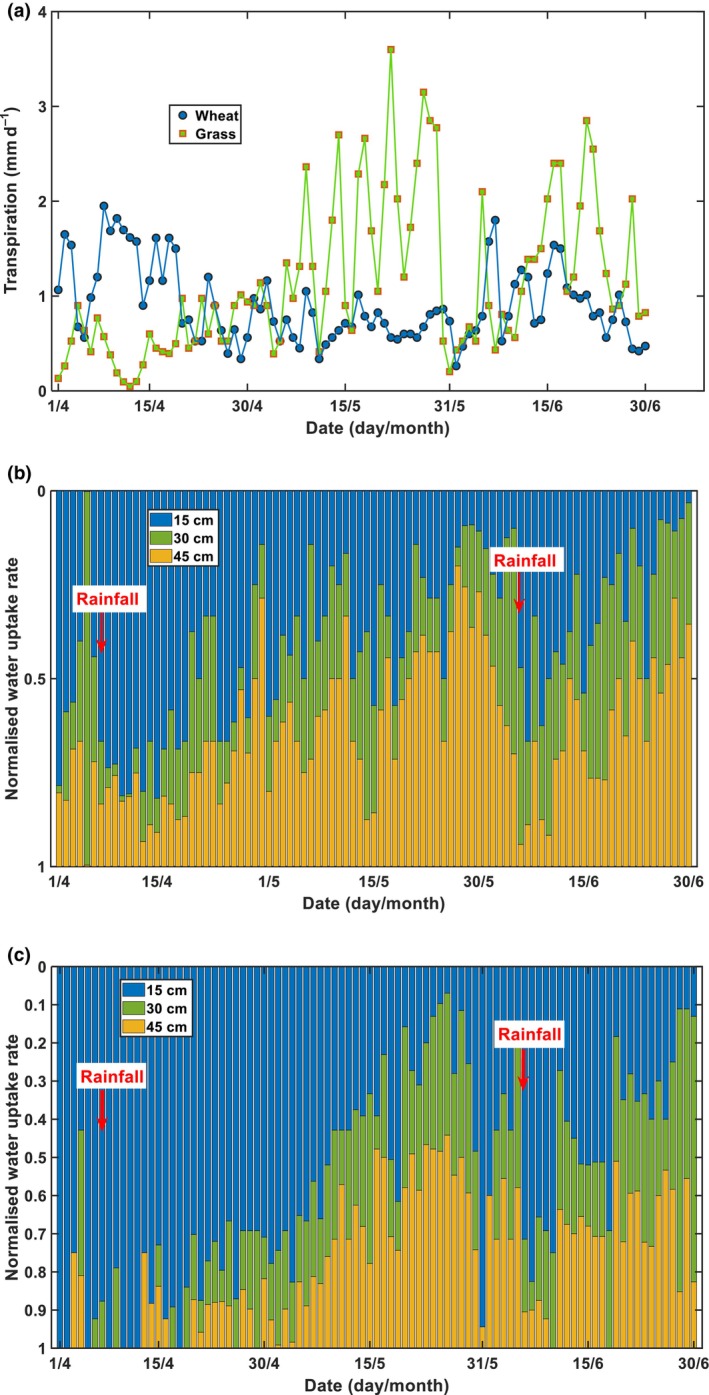
Temporal changes in total root water uptake in the two plant systems (a). Contribution of roots in different soil layers to the total root water uptake in the wheat (*Triticum aestivum*) field (b) and the grassland (c). The red arrows indicate the two rainfall events.

The spatiotemporal changes in soil water content are shown in Fig. S2. Using root water uptake in the 0–50 cm soil as an approximation of plant transpiration, we calculated it using interpolations based on daily root water uptakes measured at the three soil depths. Fig. 2(a) shows the variation in daily transpiration from 1 April to 30 June 2022 in the two plant systems, and Fig. 2(b,c) compare the contribution of each soil layer. The transpiration varied erratically (Fig. 2a), due to the influence of meteorological factors, but a consistent pattern emerged for both plant systems after normalising the daily root water uptake in each soil layer by daily transpiration: as the topsoil dried after each rainfall, the plants gradually reduced their water extraction from the topsoil, accompanied by an increase in water uptake from the subsoil (Fig. 2b,c). For example, after the rainfall in early April, water extracted by the grass roots in the 45‐cm soil layer increased from < 10% before the rainfall to 50% on 4 June before the second rainfall on 5 June. After each rainfall, both plant systems immediately increased their water uptake from the topsoil, accompanied by a decrease in water uptake from the subsoil (Fig. 2b,c).

### Changes in root water uptake with soil water content

**Fig. 3:**
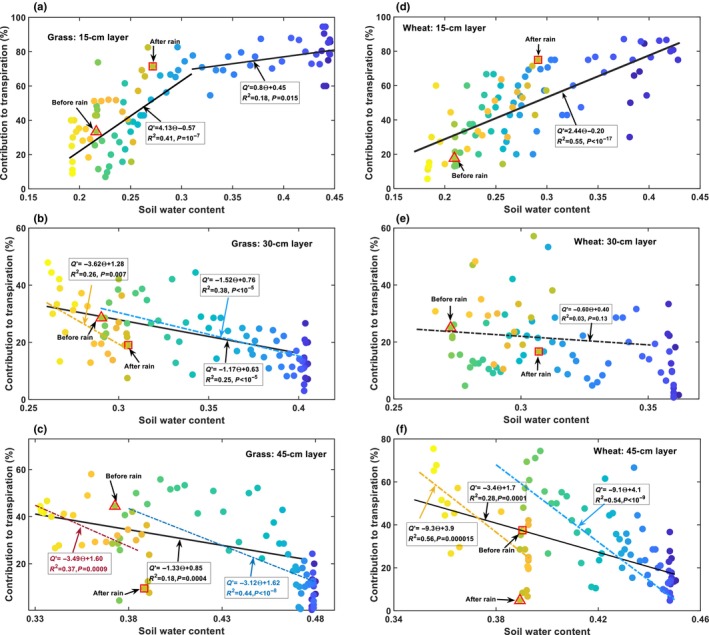
Changes in root water uptakes (normalised) with soil water content for different soil layers in the two plant systems: the grassland (a–c) and the wheat (*Triticum aestivum*) field (d–f). The colour gradients in the symbols represent dates from 1 April (dark blue) to 30 June (bright yellow). The large open triangles indicate the points just before the 5 June rainfall, and the open squares indicate the points just after the 5 June rainfall.

Root water uptake in each soil layer was influenced by meteorological factors and soil water. To ameliorate the impact of the fluctuating meteorological factors, we normalised the daily root water uptake in each layer by daily transpiration to yield a relative root water uptake (contribution of each of the soil layers) to analyse the changes in root water uptake with soil water content. Fig. 3 shows the results, where the colour gradient in the symbols represents dates from 1 April to 30 June.

In the 15‐cm soil layer, the relative daily root water uptake decreased with soil water content in both plant systems, but there was a difference between the two. In the grass system, Fig. 3(a) shows that, as the topsoil water content decreased from 44% on 1 April to 30% on 1 May, the relative root water uptake decreased slightly at a significant level with *P* = 0.015, indicating no or minor water stress. A further decrease in the topsoil water content from 1 May to 4 Jun resulted in a steep decline in relative root water uptake. The rainfall on 5 June increased the topsoil water content from 21% to 28%, leading to an immediate increase in root water uptake from the topsoil (Fig. 3a). For topsoil water content < 30%, root water extraction from the topsoil decreased with soil water content, both before and after the 5 June rainfall, significantly with *P* < 10^−7^ (Fig. 3a). In contrast to the grass system, Fig. 3(d) shows that the relative water uptake of shallow roots in the wheat system decreased with the topsoil water content, both before and after the 5 June rainfall, monotonically at significant levels with *P* < 10^−17^.

**Fig. 4:**
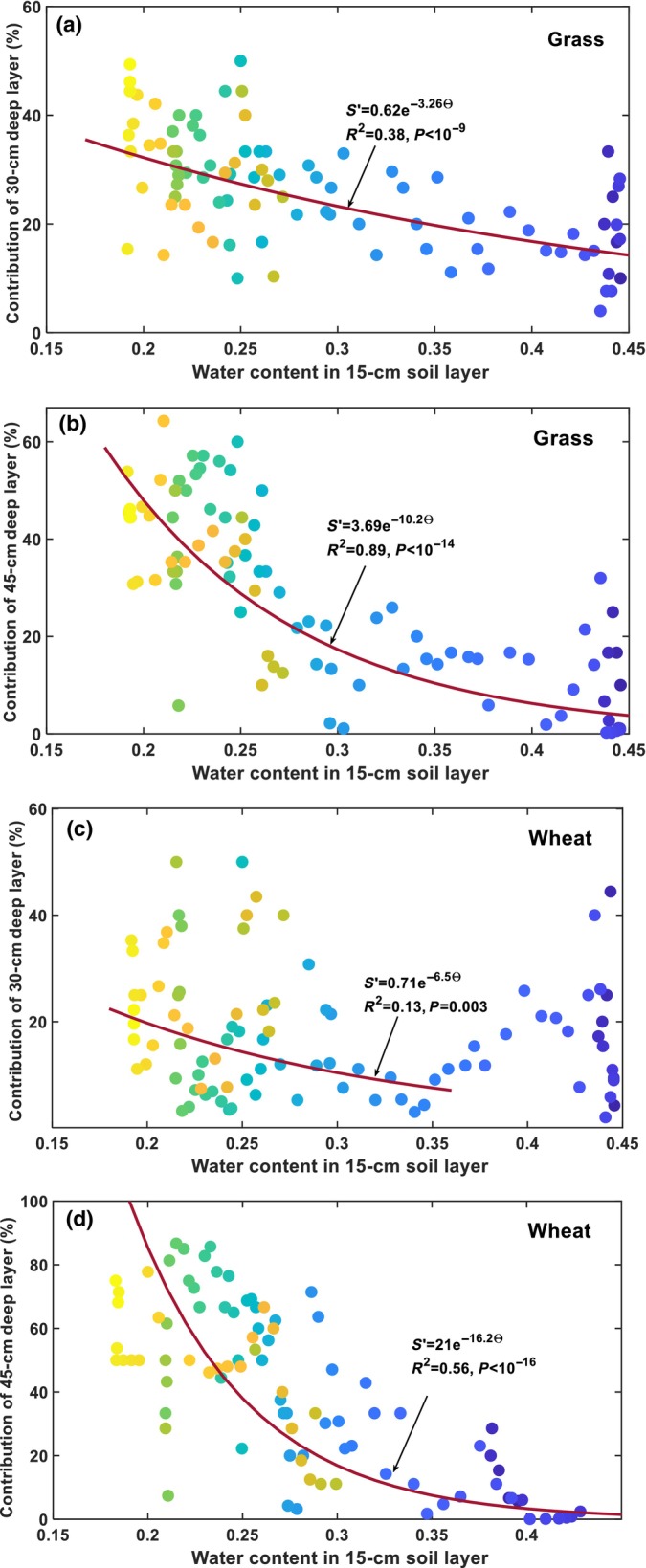
In both plant systems, root water uptakes (normalised) in the subsoil layers are correlated with the topsoil water content (15‐cm soil layer) more significantly than with the subsoil water content. The plant systems were the grassland (a, b), and the wheat (*Triticum aestivum*) field (c, d). The colour gradients in the symbols represent dates from 1 April (dark blue) to 30 June (bright yellow).

Contrary to the 15‐cm soil layer, the relative root water uptake in the 30 cm and 45 cm subsoil layers in the grass system (Fig. 3b,c) and the wheat system (Fig. 3e,f) both increased as the subsoil water content decreased from the onset of the experiment to 4 June (Fig. 2). The 5 June rainfall led to an immediate decrease in root water uptake from the two subsoil layers in both plant systems. As the topsoil water content decreased after the 5 June rainfall (Fig. 3a,d), both plant systems gradually increased their water extraction from the subsoil layers, although their water content decreased during this period (Fig. 3b,c,e,f). Overall, subsoil water content and root water uptake in the subsoil layers were correlated, but not as significantly as those in the top 15‐cm soil layer (Fig. 3a,d). In the wheat system, water content and root water uptake in the 30‐cm soil layer were not significantly correlated with *P* = 0.13 (Fig. 3e). Separating soil water content and root water uptake in the subsoil layers into a before‐rainfall (the 5 June rainfall) group and an after‐rainfall group revealed significant differences in the relationship between soil water content and root uptake in the two groups for the grass (Fig. 3b,c) and the wheat system (Fig. 3f), though the significance levels vary between soil layers and the plant systems (Fig. 3). To elucidate what coordinated root uptake from different soil layers, we analysed the correlation between relative root water uptake in the subsoil layers with the topsoil water content, the results of which are shown in Fig. 4.

**Fig. 5:**
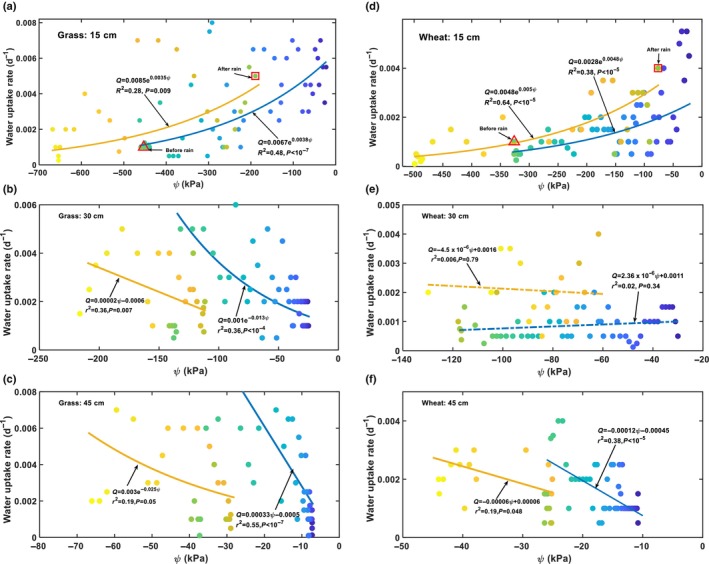
Changes in root water uptake rate (volume of water taken daily by roots in a unit volume of soil) with soil matric potential in different soil layers in the two plant systems (a–c for the grass system, and d–f for the wheat system). The colour gradients in the symbols represent dates from 15 April (dark blue) to 25 June (bright yellow). The solid lines are the best‐fitting curves. The blue lines represent the data before the 5 June rainfall, and the orange lines represent the data after the 5 June rainfall.

Water flow from soil into the xylem vessels in a root is driven by a water potential difference between them. Fig. 5 shows the changes in root water uptake rate (volume of water taken daily from a unit volume of soil) with soil matric potential in each soil layer for the two systems. We fitted soil matric potential and root water uptake before and after the 5 June rainfall into separate functions and used these functions to calculate root water potential and radial root water permeability. Despite the fluctuating meteorological factors, there is a significant correlation between root water uptake and soil matric potential in all but the 30‐cm soil layer in the wheat field.

### Root water potential and radial water permeability of shallow roots

**Fig. 6:**
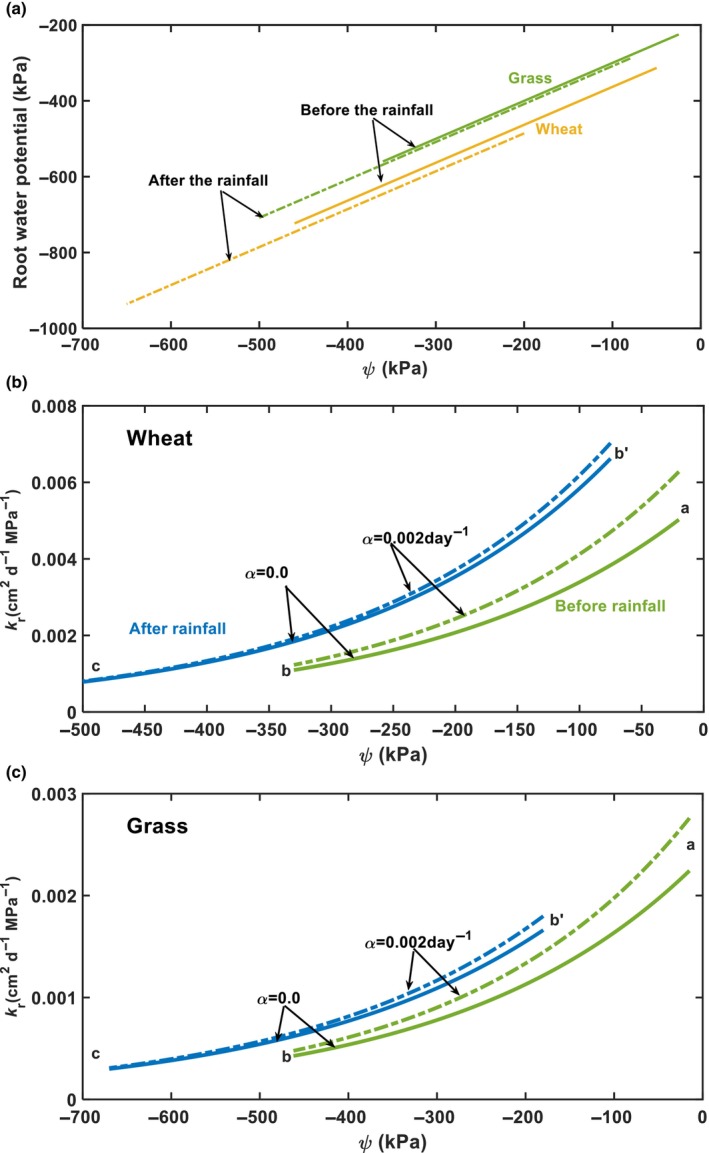
Changes in root water potential and radial root water permeability at the depth of 15 cm calculated using the relationships between root water uptake rate and soil matric potential for the two plant systems (Fig. 5(a) for the grassland and Fig. 5(d) for the wheat field). (a) Root water potential: the solid lines and dashed lines represent results calculated using data before and after the 5 June rainfall, respectively. (b, c) Radial root water permeability at the depth of 15 cm: the solid green line and the dashed green line represent the results calculated using the data before the 5 June rainfall when roots did not grow (*α* = 0) or grew at a rate of *α* = 0.002 d^−1^, respectively; the solid blue line and dashed blue line represent the results calculated using data after the 5 June rainfall when the roots did not grow (*α* = 0) or grew at a rate of *α* = 0.002 d^−1^, respectively; ‘a’ marks the onset of the experiment, ‘b’ marks the point just before the 5 June rainfall, ‘b'‘ marks the point just after the 5 June rainfall, and ‘c’ marks the end of the experiment.

Root water uptake in the top 15‐cm soil layer decreased with soil matric potential in a similar trend before and after the 5 June rainfall. Root water potential and radial root water permeability in this layer were calculated using Eqn 7. Fig. 6(a) shows the changes in root water potential with soil matric potential before and after the 5 June rainfall. Under the same soil matric potential, root water potential of the grass before and after the 5 June rainfall was close, while root water potential of the wheat after the rainfall was slightly more negative than that before the rainfall, though not significantly.

Calculating radial root water permeability requires root length and the ratio *R*_i_ : *r*_i_ for individual roots. Stele diameter is proportional to root diameter; the ratio *R*_i_ : *r*_i_ for root segments at the same soil depth can be approximated as constant. Root length densities measured at the end of the experiment are shown in the Fig. S3. Fig. 6(b,c) show the relationship between the radial root water permeability and soil matric potential, and how changes in root growth rate from *α* = 0 to *α* = 0.002 d^−1^ affect this relationship. The radial root water permeability increased with soil matric potential in both plant systems, although considering root growth gave a slightly higher root water permeability than if this was not considered. After the first rainfall in early April (marked by ‘a’ in Fig. 6b,c), both soil matric potential and radial root water permeability decreased until 4 June before the 5 June rainfall (marked by ‘b’ in Fig. 6b,c). After the 5 June rainfall (marked by ‘b'’ in Fig. 6b,c), the radial root water permeability in both plant systems increased immediately, followed by a gradual decrease as the soil matric potential decreased.

### Radial water permeability of roots in the subsoil

Except in the 30‐cm soil layer in the wheat field (Fig. 5e), root water uptake and soil matric potential were negatively correlated (Fig. 5b,c,f). These negative correlations are compensatory water uptake, where plants increased their water uptake from the subsoil when the topsoil dried. Water flow from soil to roots in the subsoil is also a hydraulic process, driven by the water potential difference between them. Such negative correlations are possible only when plants enhance their radial root water permeability or reduce their root water potential faster than the decreasing rate of soil matric potential to increase the water potential difference (*ψ* – *ψ*_0_) as shown in Eqn 6, as the soil dried.

**Fig. 7:**
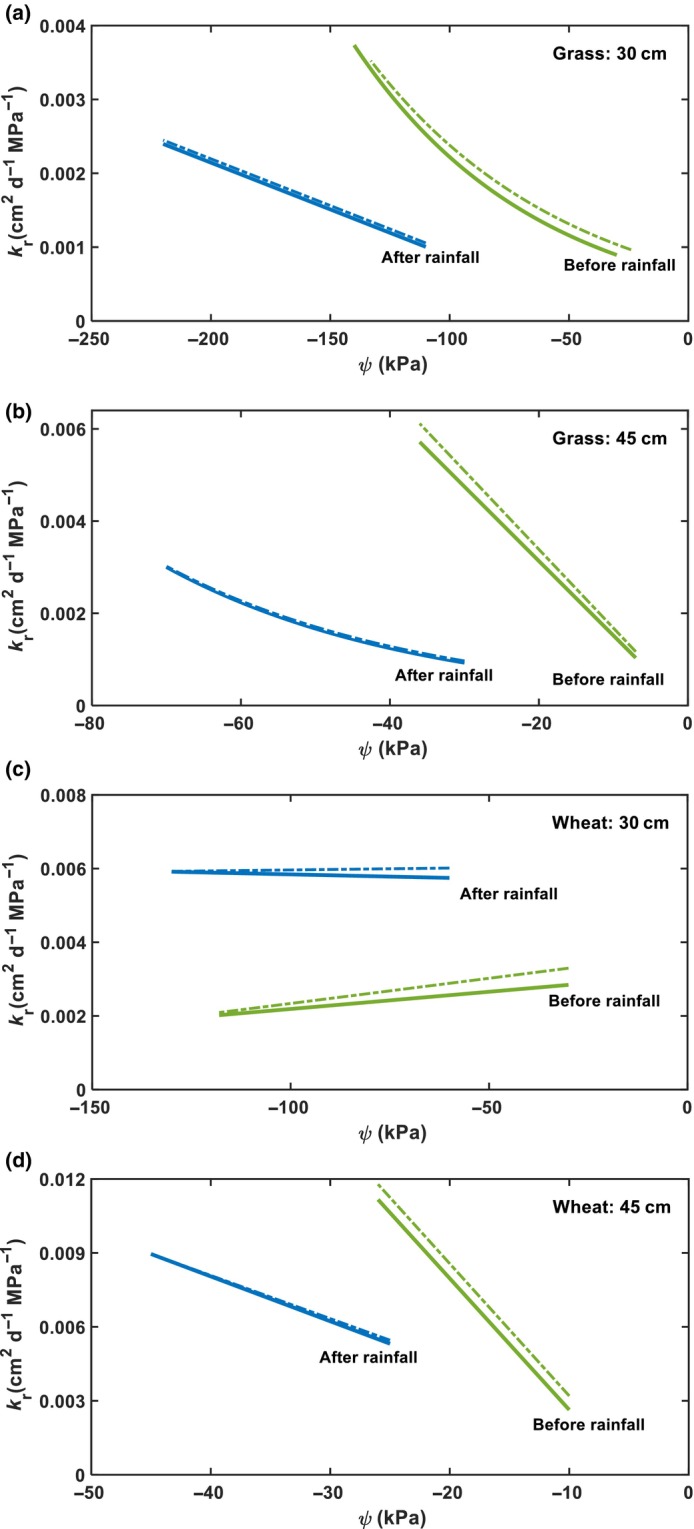
Changes in radial root water permeability with soil matric potential at the depths of 30 and 45 cm before and after the 5 June rainfall in the two plant systems ((a) and (b) for the grass system; (c) and (d) for the wheat system). The solid lines are the results calculated when roots did not grow (*α* = 0), and the associated dashed lines are the results calculated when roots grew at a rate of *α* = 0.002 d^−1^, from 1 April to 30 June.

We calculated the radial root water permeability in each subsoil layer using Eqn 8. The root water potential in the subsoil was estimated assuming that the relationship between soil matric potential and root water potential is independent of soil depth. For each soil matric potential in a subsoil layer, its associated root water potential was estimated from the curves in Fig. 6(a). Fig. 7 shows the results and how changes in root growth rate from *α* = 0 to *α* = 0.002 d^−1^ affect the calculated radial root water permeability. Except at the depth of 30 cm in the wheat field (Fig. 7c), the radial root water permeability in the two subsoil layers increased from 7 April to 4 June before the 5 June rainfall in both plant systems, even though soil water in the subsoil layers decreased in this period (Fig. S2). After the 5 June rainfall, the radial root water permeability in both subsoil layers decreased immediately in the two plant systems, followed by a gradual increase as soil water content decreased. Fig. 7 indicates that root growth only has a minor effect on the change in radial root water permeability with soil matric potential.

## Discussion

The adaptive change in root hydraulic conductivity is a hydraulic rheostat to maintain water status in the whole plant by coordinating water uptake from different soil depths (Maurel *et al*., 2010). However, the study on how plants achieve this in the field is missing (Baca Cabrera *et al*., 2024), because of the difficulties associated with *in‐situ* measurements (Boursiac *et al*., 2022b). We developed a method to calculate daily root water uptake, root water potential, and radial root water permeability using soil moisture and root‐length density measured in a wheat field and a permanent grass field (Fig. 2).

Our method cannot distinguish between soil‐surface evaporation and root water uptake. It was derived assuming that root water uptake at night is negligible (Eqn 2). We thus selected a period from 1 April to 31 June over which soil surface evaporation was negligible. April to June is the main vegetative growth season of wheat in the UK (Gregory *et al*., 1978), during which the leaf area index was > 3.6 (Zhang *et al*., 2020). We visually checked the field to ensure plant canopies fully covered the soil surface during the experimental period (Fig. S4), so that soil surface evaporation was indeed negligible compared to plant transpiration. Night transpiration was observed for some plants, but it was small, accounting for only 8–10% of the evapotranspiration (Thomas *et al*., 2020). Temporal change in topsoil water content in our experiment showed that unless soil water content was high after rainfall, topsoil water content remained approximately constant during the night (Fig. S5), indicating that night transpiration was negligible compared to daytime transpiration.

Consistent with previous findings (Teuling *et al*., 2006; Thomas *et al*., 2020), our results showed compensatory root water uptake in both plant systems, in that when the topsoil dried and shallow root water uptake decreased, the plants increased their water uptake from the subsoil (Fig. 2b,c). Shallow root water uptake and topsoil water content were positively correlated in both plant systems (Figs 3a,d, 4a,d), suggesting that topsoil water availability primarily influenced shallow root water uptake. Unexpectedly, a negative correlation was found between root water uptake and soil water content in the subsoil layers (Fig. 3b,c,e,f), especially in the 45‐cm soil layer, suggesting that the subsoil water had no influence on its uptake by the roots.

We measured root length density at the end of the experiment and used an extrapolation to evaluate the impact of root growth on the calculated root water permeability. The results calculated considering root growth at 0.002 d^−1^ are close to those calculated when this was not considered (Figs 6, 7). Previous studies have shown that in the United Kingdom, seminal wheat root growth concludes by early March and nodal roots complete their development by mid‐May (Gregory *et al*., 1978). Therefore, *α* = 0.002 d^−1^ did not underestimate root growth from 1 April to 31 June (Gregory *et al*., 1978). In the field, root growth varies with soil moisture and growing season; the results calculated with *α* = 0 and *α* = 0.002 d^−1^ can serve as an envelope for intermediate root growth rates.

We assumed the water potential difference between the bulk soil and the soil–root interface was negligible compared to the water potential difference between the soil–root interface and xylem vessels. This is supported by other research, which showed that soil–root interfacial resistance is the critical resistance determining the response of plants to water stress (Abdalla *et al*., 2021; Yang *et al*., 2023). A meta‐analysis also showed that for wheat grown in loamy soils (similar to the soil texture in our experiment), soil matric potential in the bulk soil is close to the water potential on the soil–root interface for soil matric potentials from −1000 to −10 kPa (Cai *et al*., 2022). The lowest soil matric potential in our experiment was −500 kPa in the wheat field and −800 kPa in the grassland (Fig. 5). The likely mechanisms underlying this phenomenon are that decreasing soil water potential increases suberin and lignin in the endodermis and exodermis of the roots and downregulates aquaporins (Suresh *et al*., 2024), thereby increasing root resistance to water flow. Therefore, although decreasing soil water content reduces soil hydraulic conductivity, it also reduces the radial water permeability of the root (Kreszies *et al*., 2019, 2020). Thus, the root resistance is still the dominant resistance to water flow from the soil into the xylem vessels.

Radial root water permeability was calculated assuming roots and soil were physically connected. Roots and soil could become detached when soil dries due to soil and root shrinkage (Duddek *et al*., 2022), but this was unlikely to have occurred in the soil below 15 cm depth. Minor water stress did occur in the topsoil (Fig. 3a,d), but during most of the time, the soil matric potential at the depth of 15 cm remained above −500 kPa in the wheat field and −800 kPa in the grass field. Also, the subsoils were moist (Fig. 5), and the plants did not experience droughts that reduced root water uptake. Fig. 2(a) shows that even during the driest period from 10 April to 31 May (Fig. S2), transpiration of the two plant systems did not decrease. Fig. 3 further shows that the reduced water uptake from the topsoil (Fig. 3a,d) was compensated for by the increased water uptake from the subsoil (Fig. 3b,c,e,f), suggesting root shrinkage was unlikely. While we cannot provide direct evidence to rule out soil shrinkage when soil dried, indirect evidence suggests that this is unlikely. If roots detached from the soil due to soil shrinkage when soil water content decreased to its low level before the 5 June rain (Figs 3a,d, S3), the gaps between soil and roots would be barriers for water to flow through, and root water uptake could not resume to its levels when the soil was moist, as shown in Fig. 3(a,d). Therefore, the mechanism underlying the reduced water uptake of the shallow roots when soil dried in the two plant systems was the adaptive change in root water permeability.

Mycorrhizal fungi can transport distant water into roots when soil dries (Kakouridis *et al*., 2022), but this was unlikely to be an important contributor to the increased root water uptake in the subsoil, because the majority of mycorrhizal fungi inhabit topsoil (T. Sun *et al*., 2021). Thus, the increased root water uptake in the subsoil when soil dried was the adaptive change in their radial root water permeability, consistent with the compensatory root water uptake concept (Thomas *et al*., 2020; Tzohar *et al*., 2021).

The complex interactions between soil and roots pose a significant challenge for *in situ* measurements. Approximations are thus necessary, though they might give rise to errors. Our model calculated root water uptake and root water permeability based on mass balance. It does not require soil and plant parameters and naturally captures the influence of spatiotemporal changes in soil structure induced by root‐associated activities (Rabbi *et al*., 2018). The influence of changes in soil structure and soil texture on water flow across soil sections is represented by the first term on the right‐hand side of Eqn 1, approximated by Eqn 3, and calculated by the second term on the right‐hand side of Eqn 4 through nighttime changes in soil moisture. The finer and less permeable the soil is, the smaller the changes in soil moisture at night.

The scattered data in Fig. 5 is due to meteorological factors rather than noise, as fluctuating meteorological factors and changes in soil water jointly mediated root water uptake. This is manifested by the significant correlations between root water uptake and soil water content in all soil layers after ameliorating the meteorological effect by normalising daily root water uptake in each soil layer by daily transpiration (Fig. 3). The shift from a positive correlation between root water uptake and soil matric potential at the depth of 15 cm to a negative correlation at the depth of 45 cm suggests the presence of a transition depth at which root water uptake and soil matric potential are not significantly correlated. In the wheat field, this transition depth is 30 cm (Fig. 5e); in the grass field, it is between 15 cm and 30 cm, as root water uptake and matric potential at the depth of 30 cm were significantly and negatively correlated (Fig. 5b). Such a transition depth was also observed in other plants (Müllers *et al*., 2023).

### Radial root water permeability

The radial root water permeability was calculated based on Darcy's law (Methods S2). It differs from root hydraulic conductivity used by others (Sutka *et al*., 2011; Zarebanadkouki *et al*., 2016; Heymans *et al*., 2021), where water uptake of a root segment with length *L* and radius *R* is calculated by $Q=2\pi {\mathrm{RLk}}_{R}\left(\psi -\psi_{0}\right)$. Since water flow in this context is driven by water potential difference rather than by water potential gradient, the unit of root hydraulic conductivity (*k*_R_) is cm d^−1^ MPa^−1^, when water potential is expressed as pressure (MPa). Root hydraulic conductivity depends on root radius and water permeability of the peripheral cell layers (Methods S2). Since radial root water permeability represents the ability of the peripheral cell layers to transport water, it better describes how plants regulate their root hydraulics to respond to environmental changes. For a root segment with radius R, the relationship between root hydraulic conductivity (*k*_R_) and root water permeability is $k_{R}=k_{r}/R$.

Our results show that even for the same roots at the depth of 15 cm, their radial water permeability varied by a factor of 6 (Fig. 6b,c), consistent with the findings of a meta‐analysis (Baca Cabrera *et al*., 2024). The radial root water permeability of wheat at the depth of 15 cm varied from 0.001 to 0 0.007 cm^2^ MPa^−1^ d^−1^ (Fig. 6b). Using an average root diameter of 0.6 mm (Bramley *et al*., 2009; Hobson *et al*., 2023), the associated hydraulic conductivity was from 7.7 × 10^−9^ to 5.4 × 10^−8^ m s^−1^ MPa^−1^, comparable to that measured by Bramley *et al*. (2007, 2009) using different methods for segmented roots, which varied from 5.2 × 10^−8^ to 1.84 × 10^−7^ m s^−1^ MPa^−1^. The meta‐analysis of Cabrera *et al*. (2024) showed that the average radial root hydraulic conductivity of C_3_ grasses was 5.2 × 10^−8^ m s^−1^ MPa^−1^, comparable to radial hydraulic conductivity of our grass roots (assuming a root radius is 0.02 cm) at the depths of 30 and 45 cm, which varied from 1.15 × 10^−8^ to 6.7 × 10^−8^ m s^−1^ MPa^−1^ (Fig. 7c,d).

The radial root water permeability we calculated is the average of all living root segments in a soil layer, including aged roots and transportive roots (McCormack *et al*., 2015). It is thus likely to be at the lower end of the values measured using segmented young roots.

### Root water potential

Root water potential was commonly measured by pressurising sap out of de‐topped roots (Dodd *et al*., 2008) and segmented roots (Ganesan *et al*., 2024); the response of root water potential to soil water change was measured by growing plants in pots with different soil matric potential (Dodd *et al*., 2010; Ganesan *et al*., 2024). Since root water potential is a dynamic process, changing with water flow in the xylem vessels, and plants grown in different soil water potential have different root anatomies (Scoffoni *et al*., 2017), root water potential measured in such ways is unlikely to be the same as the water potential of living roots in the field. While directly validating our results is difficult, calculating radial root water conductivity requires root water potential. The close agreement between the calculated root hydraulic conductivity and those measured using different methods indicates that the calculated root water potential is representative of that in the field.

### Dual role of the topsoil water

The negative correlation between root water uptake and soil matric potential in the subsoil (Fig. 5b,c,e,f) suggests that subsoil water had no influence on its uptake by roots. This is consistent with the experimental results of Müllers *et al*. (2023), where decreasing soil matric potential increased water uptake of maize and faba bean from the subsoil (below 15 cm depth for the faba bean and 25 cm depth for the maize). Fig. 3 shows that in both plant systems, a decrease in shallow root water uptake (Fig. 3a,d) was accompanied by an increase in root water uptake in the subsoil (Fig. 3b,c,e,f). Fig. 4 indicates root water uptake (normalised) in the two subsoil layers correlates with the topsoil water content more significantly than with the subsoil water contents for both plant systems, with the *P*‐value varying from 0.13 to 10^−9^ for the former (Fig. 3b,c,e,f) and from 0.003 to 10^−16^ for the latter (Fig. 4). This suggests that the topsoil water serves not only as a resource but also as a cue, coordinating root water uptake from the soil profile. Specifically, when the topsoil dried, the plants increased their water uptake from the subsoil, whereas when the topsoil was rewetted, the plants promptly reduced their water uptake from the subsoil, accompanied by an increase in water uptake from the topsoil. Such adaptive changes in root water uptake may arise because nutrients, such as nitrogen and phosphorus, are more abundant in the topsoil and absorbing the topsoil water is more energy‐efficient than lifting the subsoil water (Buras *et al*., 2020; Liu *et al*., 2023). It also aligns with the ‘root economy’ concept, in that, because the topsoil water is prone to evaporation, plants preferentially adsorb it, while using the subsoil water as a reserve (Wen *et al*., 2022). Physiologically, roots in the topsoil are dense; absorbing the topsoil water in unregulated manners could result in soil and root shrinkage, reducing soil hydraulic connectivity and impeding the movement of distant water into the root–soil surface (Carminati & Javaux, 2020). Evolutionarily, plants are likely to have developed strategies to optimise the use of both topsoil and subsoil water. Our results suggest that plants achieve this by spatiotemporally adjusting their radial root water permeability, coordinated by periodic changes in topsoil water (Figs 6, 7).

### Adaptive changes in radial root water permeability

Our results for the shallow roots (Fig. 6b,c) are consistent with previous experiments conducted in pots and hydroponic culture in that decreasing soil water content reduced whole‐root hydraulic conductance (Hu *et al*., 2011) and radial root hydraulic conductivity of wheat (Trillo & Fernandez, 2005). Additionally, we found that rewetting the topsoil led to an immediate increase in radial water permeability of the shallow roots (Fig. 6b,c).

Compensatory root water uptake has been well documented and experimentally proven (Simunek & Hopmans, 2009; Thomas *et al*., 2020; Müllers *et al*., 2023), in which when topsoil dries, plants increase their water uptake from the moist subsoil. In the field, the topsoil and subsoil are hydraulically connected. As the topsoil dries, the subsoil water content also decreases. Compensatory water uptake requires roots to take more water from the subsoil when the topsoil dries. Referring to Eqn 6, a negative correlation between soil matric potential and root water uptake requires $\partial Q/\partial \psi =2\mathrm{\pi L}\left(\psi -\psi_{0}\right)\partial k_{r}/\partial \psi +2\pi {\mathrm{Lk}}_{r}\partial \left(\psi -\psi_{0}\right)/\partial \psi <0$, possible only when $\partial \left(\psi -\psi_{0}\right)/\partial \psi =1-\partial \psi_{0}/\partial \psi <0$, or $\partial k_{r}/\partial \psi <0$. The first condition requires $\partial \psi_{0}/\partial \psi >1$; that is, as soil matric potential decreases, plants must decrease their root water potential faster than the decreasing rate of soil matric potential so as to increase $\psi -\psi_{0}$ and enhance water uptake. The condition $\partial k_{r}/\partial \psi <0$ requires plants to increase the radial water permeability of their roots in the subsoil to enhance water uptake when matric potential decreases. $\partial \psi_{0}/\partial \psi >1$ is physically possible but not physiologically rational, as the goal of plant hydraulic rheostat is to maintain the stability of water status in the whole plant when environmental conditions change (Maurel *et al*., 2010); it is also contrary to experimental findings (Schmidhalter, 1997). Thus, the rational mechanism underlying the negative correlation between soil matric potential and root water uptake in the subsoils is that, as the topsoil (hence the subsoil) dried, the plants increased the radial water permeability of their roots in the subsoil to enhance water uptake. This is also corroborated by findings from other plants (Johnson *et al*., 2014; Müllers *et al*., 2023). Additionally, we found that after the water stress in the topsoil was relieved, the plants increased the radial water permeability of their shallow roots to boost water uptake, while reducing the radial water permeability of their subsoil roots to limit water extraction. Such adaptive changes in radial root water permeability are efficient for optimally using water in both the topsoil and subsoil, and are aligned with the root economy concept (Wen *et al*., 2022).

Radial root water permeability is influenced by various factors, including suberization and lignification in the endodermis and exodermis (Lynch *et al*., 2014; Couvreur *et al*., 2018), but its immediate changes after rainfall in the two plant systems suggest that the suberization and lignification, which are irreversible, may play a minor role. Aquaporins, which control the cell‐to‐cell pathways and are known to respond rapidly to environmental changes (Steudle, 2000; McLean *et al*., 2011; Kaneko *et al*., 2015; Shekoofa & Sinclair, 2018; Domec *et al*., 2021; Boursiac *et al*., 2022a), are likely to play the dominant role in regulating radial root water permeability (Tournaire‐Roux *et al*., 2003; Kreszies *et al*., 2020; Suresh *et al*., 2024). Our results suggest that the topsoil water downregulates aquaporin activity in the subsoil roots while upregulating it in the shallow roots.

### Difference between the plant systems

The water potential of grass roots is higher than that of wheat roots under the same soil matric potential (Fig. 6a) because the average xylem diameter of wheat roots is larger than that of grass roots (Ouyang *et al*., 2020). Axial root hydraulic conductance increases with the fourth power of root radius. A small decrease in root xylem diameter can force the plant to increase the water potential gradient in the longitudinal axis of the roots to maintain water ascent. Since the radial root water uptake rate is proportional to the water potential difference between the root and the soil (Eqn 5), more negative water potential in the grass roots enhances water uptake under the same soil matric potential, making shallow grass roots more tolerant to water stress. When the topsoil dried, the grass roots in the subsoil increased their water uptake more quickly than the wheat roots, indicating that the grass system is more efficient in using subsoil water. This finding aligns with the results of Müllers *et al*. (2023), where maize was more efficient in using subsoil water. The average annual rainfall at our experimental site is 763.5 mm, and the soil was relatively moist during the experimental period, particularly the subsoil (Fig. 3). However, since root water uptake also depends on soil water availability, we do not know whether the relationships between root water uptake and soil matric potential that we obtained for different soil layers would alter when soil water content decreases out of the range measured in the experiment. Further work is needed.

Differences in radial root water permeability exist not only between different plant species but also among different lines of the same crop (Rishmawi *et al*., 2023). Our findings highlight the mechanisms plants use to cope with periodic water stress in the field, with broader implications: in addition to root morphology and the rhizosphere (Fradgley *et al*., 2020; Hallett *et al*., 2022), phenotyping spatiotemporal changes in radial root water permeability and root water potential are also important for screening drought‐resistant crops.

### Conclusions

We developed a method to measure and calculate spatiotemporal changes in daily root water uptake, root water potential, and radial root water permeability in different soil layers in a wheat field and a permanent grass field. In both plant systems, shallow root water uptake is positively correlated with topsoil matric potential, while root water uptake in the subsoil is negatively correlated with subsoil matric potential. Root water uptake in the subsoil is influenced by the topsoil water more than by the subsoil water, indicating that the topsoil water serves not only as a resource but also as a cue, optimising water uptake from different soil layers. As soil dried after rainfall, both plant systems decreased the radial water permeability of their shallow roots to reduce topsoil water uptake, while increasing the radial water permeability of their roots in subsoil to enhance subsoil water uptake. Conversely, when rainfall rewetted the topsoil, both plant systems immediately increased the radial water permeability of their shallow roots to increase topsoil water uptake, whereas decreasing the radial water permeability of their roots in subsoil to limit subsoil water uptake. These adaptive changes in root water permeability are strategies plants use to optimise the use of soil water. There is a difference in these strategies between the two plant systems: the grass system is more tolerant to water stress in the topsoil and more efficient in using the subsoil water. These findings have significant implications for understanding the mechanisms plants use to cope with periodic water stress and suggest that, in addition to root morphology and the rhizosphere, phenotyping the adaptive changes in root hydraulic conductivity is also crucial for developing and screening drought‐tolerant crops.

## Competing interests

None declared.

## Author contributions

WR and IH conducted the field experiments and measured the root length‐density. HVC, SJM, WRW and MJH designed the concept. XZ and WRW developed the model, executed the calculations, and wrote the manuscript. All authors contributed to the writing and editing of the manuscripts.

## Disclaimer

The New Phytologist Foundation remains neutral with regard to jurisdictional claims in maps and in any institutional affiliations.

## Supporting information


**Fig. S1** Water release curves of the soils in the two fields.
**Fig. S2** Temporal changes in soil water content at different depths in the two fields.
**Fig. S3** Root‐length density distributions in the two plant systems.
**Fig. S4** A snapshot of the experimental site on 13 May 2022 to show the plant densities.
**Fig. S5** A zoom‐in view of the soil moisture changes in daytime and nighttime.
**Methods S1** Water release curve measurement.
**Methods S2** Water flow in roots and radial root water permeability.
Please note: Wiley is not responsible for the content or functionality of any Supporting Information supplied by the authors. Any queries (other than missing material) should be directed to the *New Phytologist* Central Office.

## Data Availability

The data that support the findings of this study are available in the Supporting Information of this article (Figs S2, S3). The Excel file of data supporting Fig. 2 is available for download from: https://figshare.com/articles/dataset/Soil_moisture_data/28536032?file=52794158

## References

[nph70013-bib-0001] Abdalla M , Carminati A , Cai G , Javaux M , Ahmed MA . 2021. Stomatal closure of tomato under drought is driven by an increase in soil–root hydraulic resistance. Plant, Cell & Environment 44: 425–431. 10.1111/pce.13939 33150971

[nph70013-bib-0002] Baca Cabrera JC , Vanderborght J , Couvreur V , Behrend D , Gaiser T , Nguyen TH , Lobet G . 2024. Root hydraulic properties: an exploration of their variability across scales. Plant Direct 8: e582. 38590783 10.1002/pld3.582 PMC10999368

[nph70013-bib-0003] Bachofen C , Tumber‐Dávila SJ , Mackay DS , McDowell NG , Carminati A , Klein T , Stocker BD , Mencuccini M , Grossiord C . 2024. Tree water uptake patterns across the globe. New Phytologist 242: 1891–1910. 38649790 10.1111/nph.19762

[nph70013-bib-0004] Bartlett MK , Klein T , Jansen S , Choat B , Sack L . 2016. The correlations and sequence of plant stomatal, hydraulic, and wilting responses to drought. Proceedings of the National Academy of Sciences, USA 113: 13098–13103. 10.1073/pnas.1604088113 PMC5135344 27807136

[nph70013-bib-0005] Boursiac Y , Pradal C , Bauget F , Lucas M , Delivorias S , Godin C , Maurel C . 2022a. Phenotyping and modeling of root hydraulic architecture reveal critical determinants of axial water transport. Plant Physiology 190: 1289–1306. 35708646 10.1093/plphys/kiac281 PMC9516777

[nph70013-bib-0006] Boursiac Y , Protto V , Rishmawi L , Maurel C . 2022b. Experimental and conceptual approaches to root water transport. Plant and Soil 478: 349–370. 36277078 10.1007/s11104-022-05427-z PMC9579117

[nph70013-bib-0007] Bramley H , Turner NC , Turner DW , Tyerman SD . 2007. Comparison between gradient‐dependent hydraulic conductivities of roots using the root pressure probe: the role of pressure propagations and implications for the relative roles of parallel radial pathways. Plant, Cell & Environment 30: 861–874. 10.1111/j.1365-3040.2007.01678.x 17547657

[nph70013-bib-0008] Bramley H , Turner NC , Turner DW , Tyerman SD . 2009. Roles of morphology, anatomy, and aquaporins in determining contrasting hydraulic behavior of roots. Plant Physiology 150: 348–364. 19321713 10.1104/pp.108.134098 PMC2675714

[nph70013-bib-0009] Buras A , Rammig A , Zang CS . 2020. Quantifying impacts of the 2018 drought on European ecosystems in comparison to 2003. Biogeosciences 17: 1655–1672.

[nph70013-bib-0010] Cai GC , Ahmed MA , Abdalla M , Carminati A . 2022. Root hydraulic phenotypes impacting water uptake in drying soils. Plant, Cell & Environment 45: 650–663. 10.1111/pce.14259 PMC9303794 35037263

[nph70013-bib-0011] Carminati A , Javaux M . 2020. Soil rather than xylem vulnerability controls stomatal response to drougnt. Trends in Plant Science 25: 868–880. 32376085 10.1016/j.tplants.2020.04.003

[nph70013-bib-0012] Clarkson DT , Carvajal M , Henzler T , Waterhouse RN , Smyth AJ , Cooke DT , Steudle E . 2000. Root hydraulic conductance: diurnal aquaporin expression and the effects of nutrient stress. Journal of Experimental Botany 51: 61–70. 10938796

[nph70013-bib-0013] Corso D , Delzon S , Lamarque LJ , Cochard H , Torres‐Ruiz JM , King A , Brodribb T . 2020. Neither xylem collapse, cavitation, or changing leaf conductance drive stomatal closure in wheat. Plant, Cell & Environment 43: 854–865. 10.1111/pce.13722 31953855

[nph70013-bib-0014] Couvreur V , Faget M , Lobet G , Javaux M , Chaumont F , Draye X . 2018. Going with the flow: multiscale insights into the composite nature of water transport in roots. Plant Physiology 178: 1689–1703. 30366980 10.1104/pp.18.01006 PMC6288756

[nph70013-bib-0015] Couvreur V , Vanderborght J , Javaux M . 2012. A simple three‐dimensional macroscopic root water uptake model based on the hydraulic architecture approach. Hydrology and Earth System Sciences 16: 2957–2971.

[nph70013-bib-0016] Davies WJ , Bennett MJ . 2015. Achieving more crop per drop. Nature Plants 1: 2. 10.1038/nplants.2015.118 27250549

[nph70013-bib-0017] Deseano Diaz PA , van Dusschoten D , Kübert A , Brüggemann N , Javaux M , Merz S , Vanderborght J , Vereecken H , Dubbert M , Rothfuss Y . 2023. Response of a grassland species to dry environmental conditions from water stable isotopic monitoring: no evident shift in root water uptake to wetter soil layers. Plant and Soil 482: 491–512.

[nph70013-bib-0018] Dodd IC , Egea G , Davies WJ . 2008. Abscisic acid signalling when soil moisture is heterogeneous: decreased photoperiod sap flow from drying roots limits abscisic acid export to the shoots. Plant, Cell & Environment 31: 1263–1274. 10.1111/j.1365-3040.2008.01831.x 18507805

[nph70013-bib-0019] Dodd IC , Egea G , Watts CW , Whalley WR . 2010. Root water potential integrates discrete soil physical properties to influence ABA signalling during partial rootzone drying. Journal of Experimental Botany 61: 3543–3551. 20591896 10.1093/jxb/erq195

[nph70013-bib-0020] Domec JC , King JS , Carmichael MJ , Overby AT , Wortemann R , Smith WK , Miao GF , Noormets A , Johnson DM . 2021. Aquaporins, and not changes in root structure, provide new insights into physiological responses to drought, flooding, and salinity. Journal of Experimental Botany 72: 4489–4501. 33677600 10.1093/jxb/erab100

[nph70013-bib-0021] Draye X , Kim Y , Lobet G , Javaux M . 2010. Model‐assisted integration of physiological and environmental constraints affecting the dynamic and spatial patterns of root water uptake from soils. Journal of Experimental Botany 61: 2145–2155. 20453027 10.1093/jxb/erq077

[nph70013-bib-0022] Duddek P , Carminati A , Koebernick N , Ohmann L , Lovric G , Delzon S , Rodriguez‐Dominguez CM , King A , Ahmed MA . 2022. The impact of drought‐induced root and root hair shrinkage on root–soil contact. Plant Physiology 189: 1232–1236. 35325215 10.1093/plphys/kiac144 PMC9237671

[nph70013-bib-0023] Fradgley N , Evans G , Biernaskie JM , Cockram J , Marr EC , Oliver AG , Ober E , Jones H . 2020. Effects of breeding history and crop management on the root architecture of wheat. Plant and Soil 452: 587–600. 32713967 10.1007/s11104-020-04585-2 PMC7371663

[nph70013-bib-0024] Ganesan SP , Boldrin D , Leung AK . 2024. A closer look at root water potential: experimental evidence based on drought stress of *Chrysopogon zizanioides* . Plant and Soil 499: 569–585.

[nph70013-bib-0025] Gao W , Hodgkinson L , Jin K , Watts CW , Ashton RW , Shen J , Ren T , Dodd IC , Binley A , Phillips AL *et al*. 2016. Deep roots and soil structure. Plant, Cell & Environment 39: 1662–1668. 10.1111/pce.12684 PMC4950291 26650587

[nph70013-bib-0026] Gao Y , Yang ZJ , Wang GS , Sun JS , Zhang XX . 2020. Discerning the difference between lumens and scalariform perforation plates in impeding water flow in single xylem vessels and vessel networks in cotton. Frontiers in Plant Science 11: 12. 32211002 10.3389/fpls.2020.00246 PMC7076184

[nph70013-bib-0027] Gessler A , Bachli L , Freund ER , Treydte K , Schaub M , Haeni M , Weiler M , Seeger S , Marshall J , Hug C *et al*. 2022. Drought reduces water uptake in beech from the drying topsoil, but no compensatory uptake occurs from deeper soil layers. New Phytologist 233: 194–206. 34610146 10.1111/nph.17767 PMC9293437

[nph70013-bib-0028] Gregory AS , Dungait JAJ , Watts CW , Bol R , Dixon ER , White RP , Whitmore AP . 2016. Long‐term management changes topsoil and subsoil organic carbon and nitrogen dynamics in a temperate agricultural system. European Journal of Soil Science 67: 421–430. 27478400 10.1111/ejss.12359 PMC4950136

[nph70013-bib-0029] Gregory PJ , McGowan M , Biscoe PV , Hunter B . 1978. Water relations of winter wheat. 1. Growth of root system. Journal of Agricultural Science 91: 91–102.

[nph70013-bib-0030] Hallett PD , Marin M , Bending GD , George TS , Collins CD , Otten W . 2022. Building soil sustainability from root‐soil interface traits. Trends in Plant Science 27: 688–698. 35168900 10.1016/j.tplants.2022.01.010

[nph70013-bib-0031] Heymans A , Couvreur V , Lobet G . 2021. Combining cross‐section images and modeling tools to create high‐resolution root system hydraulic atlases in *Zea mays* . Plant Direct 5: e00290. 10.1002/pld3.334 PMC8320656 34355112

[nph70013-bib-0032] Hobson DJ , Harty MA , Langton D , McDonnell K , Tracy SR . 2023. The establishment of winter wheat root system architecture in field soils: the effect of soil type on root development in a temperate climate. Soil Use and Management 39: 198–208. 37033407 10.1111/sum.12795 PMC10078784

[nph70013-bib-0033] Hodgkinson L , Dodd IC , Binley A , Ashton RW , White RP , Watts CW , Whalley WR . 2017. Root growth in field‐grown winter wheat: some effects of soil conditions, season and genotype. European Journal of Agronomy 91: 74–83. 29129966 10.1016/j.eja.2017.09.014 PMC5669304

[nph70013-bib-0034] Hopmans JW , Bristow KL . 2002. Current capabilities and future needs of root water and nutrient uptake modeling. In: Sparks DL , ed. Advances in agronomy, vol. 77. San Diego, CA, USA: Elsevier Academic Press Inc, 103–183.

[nph70013-bib-0035] Hu T , Kang S , Li F , Zhang J . 2011. Effects of partial root‐zone irrigation on hydraulic conductivity in the soil–root system of maize plants. Journal of Experimental Botany 62: 4163–4172. 21527627 10.1093/jxb/err110 PMC3153675

[nph70013-bib-0036] Johnson DM , Sherrard ME , Domec JC , Jackson RB . 2014. Role of aquaporin activity in regulating deep and shallow root hydraulic conductance during extreme drought. Trees‐Structure and Function 28: 1323–1331.

[nph70013-bib-0037] Kakouridis A , Hagen JA , Kan MP , Mambelli S , Feldman LJ , Herman DJ , Weber PK , Pett‐Ridge J , Firestone MK . 2022. Routes to roots: direct evidence of water transport by arbuscular mycorrhizal fungi to host plants. New Phytologist 236: 210–221. 35633108 10.1111/nph.18281 PMC9543596

[nph70013-bib-0038] Kaneko T , Horie T , Nakahara Y , Tsuji N , Shibasaka M , Katsuhara M . 2015. Dynamic regulation of the root hydraulic conductivity of barley plants in response to salinity/osmotic stress. Plant and Cell Physiology 56: 875–882. 25634964 10.1093/pcp/pcv013

[nph70013-bib-0039] Kreszies T , Eggels S , Kreszies V , Osthoff A , Shellakkutti N , Baldauf JA , Zeisler‐Diehl VV , Hochholdinger F , Ranathunge K , Schreiber L . 2020. Seminal roots of wild and cultivated barley differentially respond to osmotic stress in gene expression, suberization, and hydraulic conductivity. Plant, Cell & Environment 43: 344–357. 10.1111/pce.13675 31762057

[nph70013-bib-0040] Kreszies T , Shellakkutti N , Osthoff A , Yu P , Baldauf JA , Zeisler‐Diehl VV , Ranathunge K , Hochholdinger F , Schreiber L . 2019. Osmotic stress enhances suberization of apoplastic barriers in barley seminal roots: analysis of chemical, transcriptomic and physiological responses. New Phytologist 221: 180–194. 30055115 10.1111/nph.15351 PMC6586163

[nph70013-bib-0041] Kühnhammer K , Kübert A , Brüggemann N , Deseano Diaz P , van Dusschoten D , Javaux M , Merz S , Vereecken H , Dubbert M , Rothfuss Y . 2020. Investigating the root plasticity response of *Centaurea jacea* to soil water availability changes from isotopic analysis. New Phytologist 226: 98–110. 31792975 10.1111/nph.16352

[nph70013-bib-0042] Kulmatiski A , Beard KH . 2013. Root niche partitioning among grasses, saplings, and trees measured using a tracer technique. Oecologia 171: 25–37. 22752210 10.1007/s00442-012-2390-0

[nph70013-bib-0043] Li Y , Fuchs M , Cohen S , Cohen Y , Wallach R . 2002. Water uptake profile response of corn to soil moisture depletion. Plant, Cell & Environment 25: 491–500.

[nph70013-bib-0044] Liu Z , Ye L , Jiang J , Liu R , Xu Y , Jia G . 2023. Increased uptake of deep soil water promotes drought resistance in mixed forests. Plant, Cell & Environment 46: 3218–3228. 10.1111/pce.14642 37287350

[nph70013-bib-0045] Loepfe L , Martinez‐Vilalta J , Pinol J , Mencuccini M . 2007. The relevance of xylem network structure for plant hydraulic efficiency and safety. Journal of Theoretical Biology 247: 788–803. 17509617 10.1016/j.jtbi.2007.03.036

[nph70013-bib-0046] Lynch JP . 2013. Steep, cheap and deep: an ideotype to optimize water and N acquisition by maize root systems. Annals of Botany 112: 347–357. 23328767 10.1093/aob/mcs293 PMC3698384

[nph70013-bib-0047] Lynch JP . 2019. Root phenotypes for improved nutrient capture: an underexploited opportunity for global agriculture. New Phytologist 223: 548–564. 30746704 10.1111/nph.15738

[nph70013-bib-0048] Lynch JP , Chimungu JG , Brown KM . 2014. Root anatomical phenes associated with water acquisition from drying soil: targets for crop improvement. Journal of Experimental Botany 65: 6155–6166. 24759880 10.1093/jxb/eru162

[nph70013-bib-0049] Maurel C , Nacry P . 2020. Root architecture and hydraulics converge for acclimation to changing water availability. Nature Plants 6: 744–749. 32601421 10.1038/s41477-020-0684-5

[nph70013-bib-0050] Maurel C , Simonneau T , Sutka M . 2010. The significance of roots as hydraulic rheostats. Journal of Experimental Botany 61: 3191–3198. 20522526 10.1093/jxb/erq150

[nph70013-bib-0051] McCormack ML , Dickie IA , Eissenstat DM , Fahey TJ , Fernandez CW , Guo D , Helmisaari H‐S , Hobbie EA , Iversen CM , Jackson RB *et al*. 2015. Redefining fine roots improves understanding of below‐ground contributions to terrestrial biosphere processes. New Phytologist 207: 505–518. 25756288 10.1111/nph.13363

[nph70013-bib-0052] McLean EH , Ludwig M , Grierson PF . 2011. Root hydraulic conductance and aquaporin abundance respond rapidly to partial root‐zone drying events in a riparian Melaleuca species. New Phytologist 192: 664–675. 21848988 10.1111/j.1469-8137.2011.03834.x

[nph70013-bib-0053] Müllers Y , Postma JA , Poorter H , Kochs J , Pflugfelder D , Schurr U , van Dusschoten D . 2022. Shallow roots of different crops have greater water uptake rates per unit length than deep roots in well‐watered soil. Plant and Soil 481: 475–493.

[nph70013-bib-0054] Müllers Y , Postma JA , Poorter H , van Dusschoten D . 2023. Deep‐water uptake under drought improved due to locally increased root conductivity in maize, but not in faba bean. Plant, Cell & Environment 46: 2046–2060. 10.1111/pce.14587 36942406

[nph70013-bib-0055] Ouyang W , Yin X , Yang J , Struik PC . 2020. Comparisons with wheat reveal root anatomical and histochemical constraints of rice under water‐deficit stress. Plant and Soil 452: 547–568.

[nph70013-bib-0056] Prechsl UE , Burri S , Gilgen AK , Kahmen A , Buchmann N . 2015. No shift to a deeper water uptake depth in response to summer drought of two lowland and sub‐alpine C_3_−grasslands in Switzerland. Oecologia 177: 97–111. 25273953 10.1007/s00442-014-3092-6

[nph70013-bib-0057] Rabbi SMF , Tighe MK , Flavel RJ , Kaiser BN , Guppy CN , Zhang XX , Young IM . 2018. Plant roots redesign the rhizosphere to alter the three‐dimensional physical architecture and water dynamics. New Phytologist 219: 542–550. 29774952 10.1111/nph.15213

[nph70013-bib-0058] Rasmussen CR , Thorup‐Kristensen K , Dresbøll DB . 2020. Uptake of subsoil water below 2 m fails to alleviate drought response in deep‐rooted Chicory (*Cichorium intybus* L.). Plant and Soil 446: 275–290.

[nph70013-bib-0059] Rishmawi L , Bauget F , Protto V , Bauland C , Nacry P , Maurel C . 2023. Natural variation of maize root hydraulic architecture underlies highly diverse water uptake capacities. Plant Physiology 192: 2404–2418. 37052178 10.1093/plphys/kiad213 PMC10315320

[nph70013-bib-0060] Schmidhalter U . 1997. The gradient between pre‐dawn rhizoplane and bulk soil matric potentials, and its relation to the pre‐dawn root and leaf water potentials of four species. Plant, Cell & Environment 20: 953–960.

[nph70013-bib-0061] Scoffoni C , Albuquerque C , Brodersen CR , Townes SV , John GP , Cochard H , Buckley TN , McElrone AJ , Sack L . 2017. Leaf vein xylem conduit diameter influences susceptibility to embolism and hydraulic decline. New Phytologist 213: 1076–1092. 27861926 10.1111/nph.14256

[nph70013-bib-0062] Shekoofa A , Sinclair TR . 2018. Aquaporin activity to improve crop drought tolerance. Cells 7: 10. 30158445 10.3390/cells7090123 PMC6162707

[nph70013-bib-0063] Simunek J , Hopmans JW . 2009. Modeling compensated root water and nutrient uptake. Ecological Modelling 220: 505–521.

[nph70013-bib-0064] Steudle E . 2000. Water uptake by roots: effects of water deficit. Journal of Experimental Botany 51: 1531–1542. 11006304 10.1093/jexbot/51.350.1531

[nph70013-bib-0065] Steudle E , Peterson CA . 1998. How does water get through roots? Journal of Experimental Botany 49: 775–788.

[nph70013-bib-0066] Sun Q , Gilgen AK , Signarbieux C , Klaus VH , Buchmann N . 2021. Cropping systems alter hydraulic traits of barley but not pea grown in mixture. Plant, Cell & Environment 44: 2912–2924. 10.1111/pce.14054 33763869

[nph70013-bib-0067] Sun T , Wang Y , Lucas‐Borja ME , Jing X , Feng W . 2021. Divergent vertical distributions of microbial biomass with soil depth among groups and land uses. Journal of Environmental Management 292: 112755. 33992868 10.1016/j.jenvman.2021.112755

[nph70013-bib-0068] Suresh K , Bhattacharyya S , Carvajal J , Ghosh R , Zeisler‐Diehl VV , Böckem V , Nagel KA , Wojciechowski T , Schreiber L . 2024. Effects of water stress on apoplastic barrier formation in soil grown roots differ from hydroponically grown roots: histochemical, biochemical and molecular evidence. Plant, Cell & Environment 47: 4917–4931. 10.1111/pce.15067 39110071

[nph70013-bib-0069] Sutka M , Li G , Boudet J , Boursiac Y , Doumas P , Maurel C . 2011. Natural variation of root hydraulics in arabidopsis grown in normal and salt‐stressed conditions. Plant Physiology 155: 1264–1276. 21212301 10.1104/pp.110.163113 PMC3046584

[nph70013-bib-0070] Tardieu F , Zhang J , Davies WJ . 1992. Water information is conveyed by an ABA signal from maize roots in drying field soil. Plant, Cell & Environment 15: 185–191.

[nph70013-bib-0071] Teuling AJ , Uijlenhoet R , Hupet F , Troch PA . 2006. Impact of plant water uptake strategy on soil moisture and evapotranspiration dynamics during drydown. Geophysical Research Letters 33: L03401.

[nph70013-bib-0072] Thomas A , Yadav BK , Simunek J . 2020. Root water uptake under heterogeneous soil moisture conditions: an experimental study for unraveling compensatory root water uptake and hydraulic redistribution. Plant and Soil 457: 421–435.

[nph70013-bib-0073] Tournaire‐Roux C , Sutka M , Javot H , Gout E , Gerbeau P , Luu DT , Bligny R , Maurel C . 2003. Cytosolic pH regulates root water transport during anoxic stress through gating of aquaporins. Nature 425: 393–397. 14508488 10.1038/nature01853

[nph70013-bib-0074] Trillo N , Fernandez RJ . 2005. Wheat plant hydraulic properties under prolonged experimental drought: stronger decline in root‐system conductance than in leaf area. Plant and Soil 277: 277–284.

[nph70013-bib-0075] Tzohar D , Moshelion M , Ben‐Gal A . 2021. Compensatory hydraulic uptake of water by tomato due to variable root‐zone salinity. Vadose Zone Journal 20: 14.

[nph70013-bib-0076] Vadez V . 2014. Root hydraulics: the forgotten side of roots in drought adaptation. Field Crops Research 165: 15–24.

[nph70013-bib-0077] Wen Z , White PJ , Shen J , Lambers H . 2022. Linking root exudation to belowground economic traits for resource acquisition. New Phytologist 233: 1620–1635. 34761404 10.1111/nph.17854

[nph70013-bib-0078] White RG , Kirkegaard JA . 2010. The distribution and abundance of wheat roots in a dense, structured subsoil – implications for water uptake. Plant, Cell & Environment 33: 133–148. 10.1111/j.1365-3040.2009.02059.x 19895403

[nph70013-bib-0079] Yang Y , Ma X , Yan L , Li Y , Wei S , Teng Z , Zhang H , Tang W , Peng S , Li Y . 2023. Soil–root interface hydraulic conductance determines responses of photosynthesis to drought in rice and wheat. Plant Physiology 194: 376–390. 37706538 10.1093/plphys/kiad498

[nph70013-bib-0080] Zarebanadkouki M , Meunier F , Couvreur V , Cesar J , Javaux M , Carminati A . 2016. Estimation of the hydraulic conductivities of lupine roots by inverse modelling of high‐resolution measurements of root water uptake. Annals of Botany 118: 853–864. 27539602 10.1093/aob/mcw154 PMC5055639

[nph70013-bib-0081] Zhang J , Davies WJ . 1987. Increased synthesis of ABA in partially dehydrated roots: tips and ABA transport from roots to leaves. Journal of Experimental Botany 38: 2015–2023.

[nph70013-bib-0082] Zhang XX , Whalley PA , Ashton RW , Evans J , Hawkesford MJ , Griffiths S , Huang ZD , Zhou H , Mooney SJ , Whalley WR . 2020. A comparison between water uptake and root length density in winter wheat: effects of root density and rhizosphere properties. Plant and Soil 451: 345–356. 32848280 10.1007/s11104-020-04530-3 PMC7437669

[nph70013-bib-0083] Zhu W , Zhao D , Di N , Li D , Zhou O , Sun Y , Jia L , Ding C , Xi B . 2024. Matching root water uptake patterns to fine root and soil water distributions. Plant and Soil 495: 499–516.

