# Supplementary material for: Field plants strategically regulate water uptake from different soil depths by spatiotemporally adjusting their radial root hydraulic conductivity
Source: New Phytol. 2025 Mar 19;247(2):546–61. doi: 10.1111/nph.70013 (PMC12177284; doi:10.1111/nph.70013)
Supplement: Supplementary file 1 — Fig. S1 Water release curves of the soils in the two fields. Fig. S2 Temporal changes in soil water content at different depths in the two fields. Fig. S3 Root‐length density distributions in the two plant systems. Fig. S4 A snapshot of the experimental site on 13 May 2022 to show the plant densities. Fig. S5 A zoom‐in view of the soil moisture changes in daytime and nighttime. Methods S1 Water release curve measurement. Methods S2 Water flow in roots and radial root water permeability. Please note: Wiley is not responsible for the content or functionality of any Supporting Information supplied by the authors. Any queries (other than missing material) should be directed to the New Phytologist Central Office. [file NPH-247-546-s001.pdf]

***New Phytologist* Supporting Information**

Article title: **Field plants strategically regulate water uptake from different soil depths by spatiotemporally adjusting their radial root hydraulic conductivity**

Authors: William Rickard, Imrul Hossain, Xiaoxian Zhang, Hannah V. Cooper, Sacha J, Mooney, Malcolm J. Hawkesford, W. Richard Whalley

Article acceptance date: 23 January 2025

The following Supporting Information is available for this article:

Figure S1 Water release curves of the soils in the two fields.

Figure S2 Temporal changes in soil water content at different depths in the two fields.

Figure. S3. Root-length density distributions in the two plant systems.

Figure. S4 A snapshot of the experimental site on 13 May 2022 to show the plant densities.

Figure S5 A zoom-in view of the soil moisture changes in daytime and nighttime

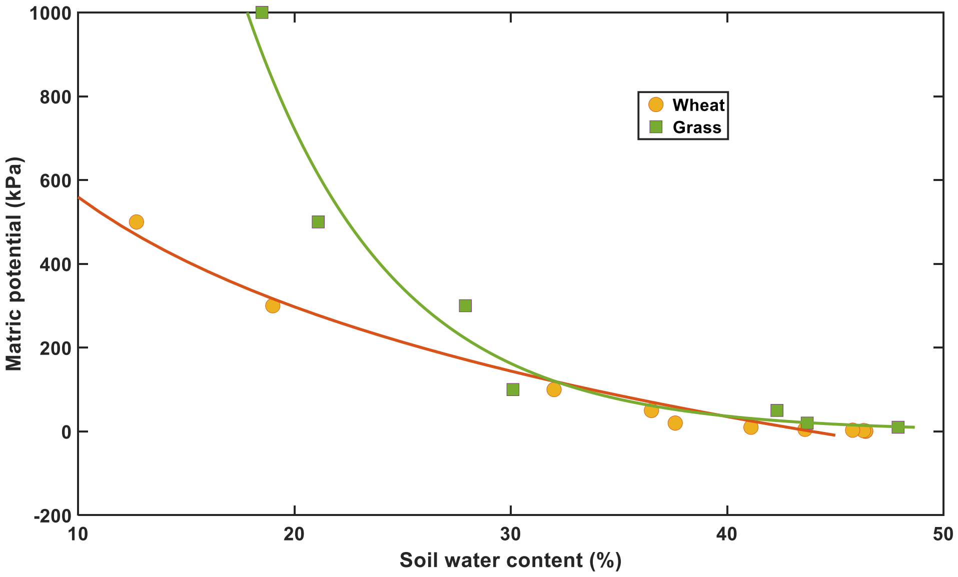

Figure S1 Water release curves of the soils in the wheat (*Triticum aestivum L.*) field and the grassland dominated by ryegrass (*Lolium pyrene L.*).

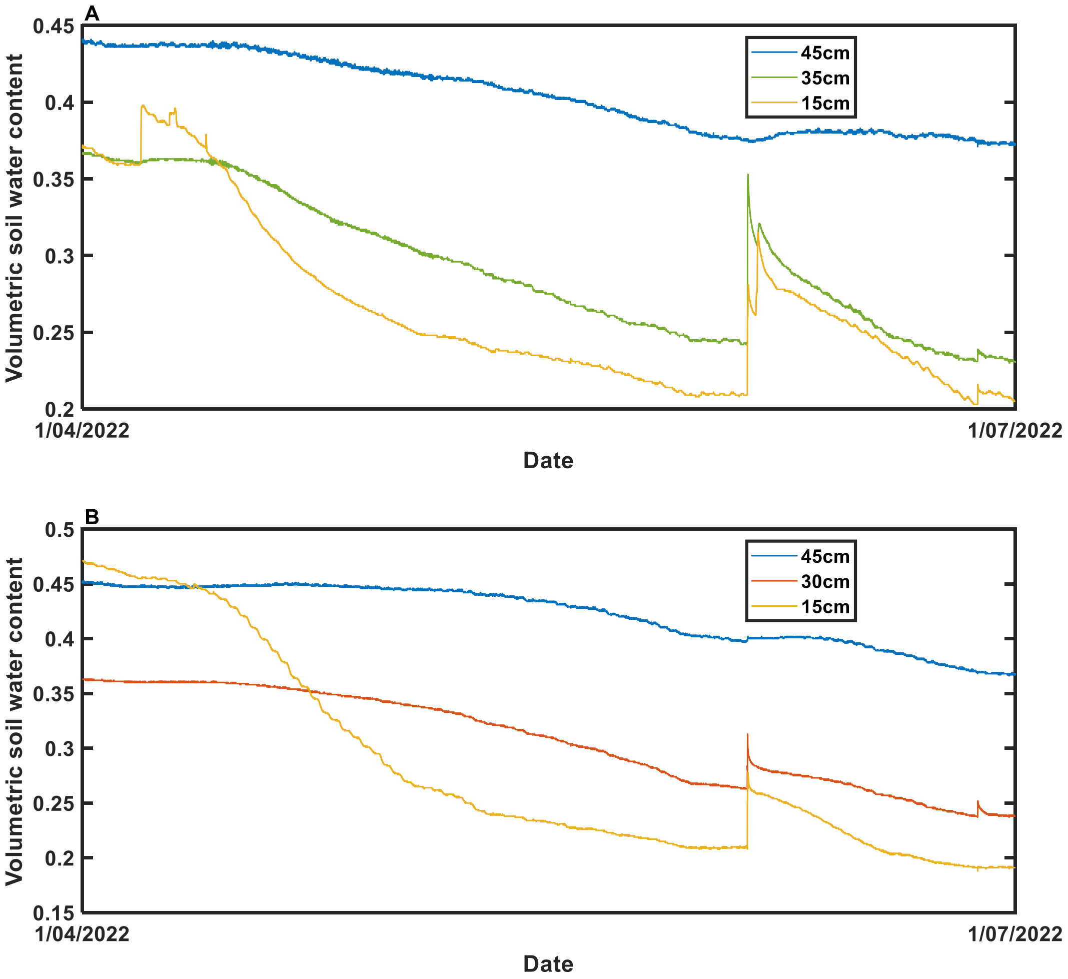

Figure S2 Temporal changes in volumetric soil water content at different depths in the wheat (*Triticum aestivum L.*) field (A) and the grassland dominated by ryegrass (*Lolium perenne L.*). (B).

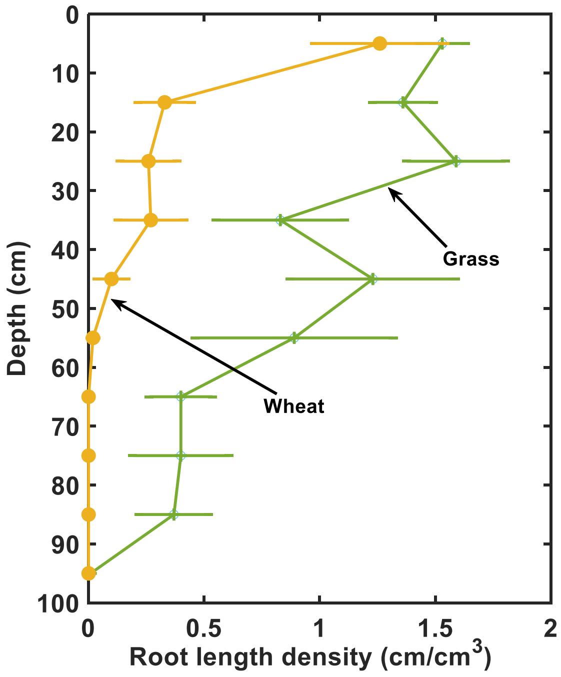

Figure S3 Root-length density distributions. The error bars represent SE).

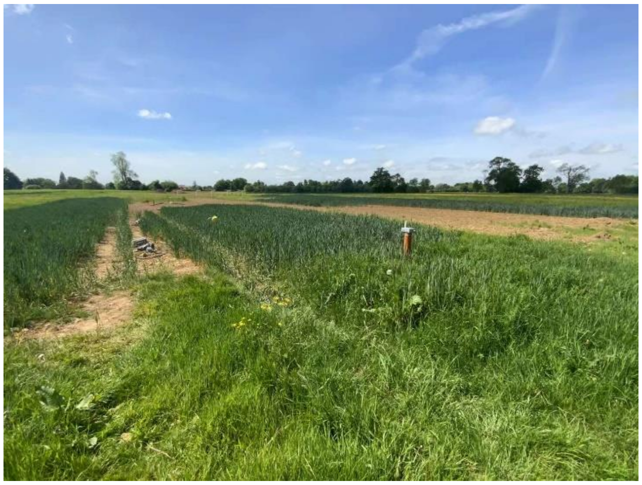

Figure S4 A snapshot of the experimental site on 13 May 2022 to show the plant densities.

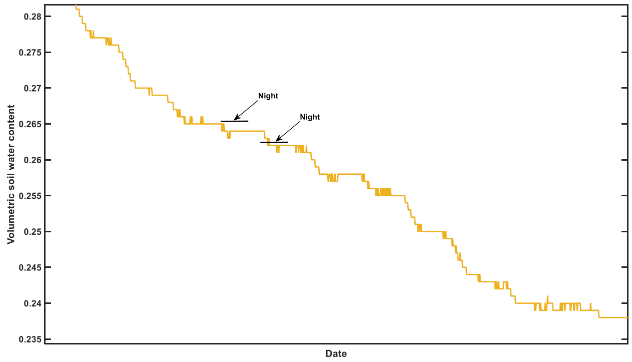

Figure S5 A zoom-in view of the soil moisture changes in daytime and nighttime.

Method S1 Water release curve measurement

Soil samples were collected from sites in the proximity of where the soil moisture sensors were installed using rings 2.5 cm high with a diameter of 5 cm. A mixture of plaster of Paris prepared at a 1 : 1 ratio was poured onto the base of the rings. For the wet end of the water release curve (from -30 to - 0.5 kPa), the rings were placed on a ceramic plate tower equipped with a hanging water column. Measurements were taken at designated heights ranging from 0 to 300 cm. For the dry end of the water release curve (from -1500 to -100 kPa), the soils were air-dried for two weeks, sieved through a 2mm sieve, and were then subjected to different pressures ranging from -15 to -1 Bar using a soil moisture ceramic plate extractor; in the measurements under each pressure, the soil samples were left for six weeks to achieve a new equilibrium. Since the volumetric soil moisture in the fields varied from more than 0.1 to 0. 45, we only used the measured water release curves for soil water content in this range. We fitted the measured soil matric potential and soil water content from all soil samples into smooth curves (Fig. S1). These smooth curves were used to calculate the matric potential associated with the soil water content measured using the soil moisture sensors.

Method S2 Water flow in roots and radial root water permeability

Water flow from the root-soil surface into xylem vessels in the root stele is driven by a water potential gradient. Since compressibility of liquid water is negligible, for a single root

segment with length L_i at soil depth z, water flow across the peripheral cell layers from the root-soil surface into the xylem vessels in the stele is described by the following equation:

$$q_i = -2\pi r k_i \frac{\partial \psi}{\partial r}, \quad Q_i = L_i \cdot q_i \qquad \text{(S1)}$$

where q_i is water flow rate across a unit length of the root segment, ψ is water potential in the perirectal cell layers at a location *r* away from the root centre (Fig. N1), k_i is the intrinsic radial water permeability of the root segment: the ability of the peripheral cell layers (including cortex, epidermis, and endodermis) to transport water. Fig. N1 shows illustratively the cross section of a root segment, in which the root radius and stele radius are R_i and r_i, respectively. Integrating Eq. (S1) from r_i and R_i yields

$$\int_{r_i}^{R_i} \frac{q_i}{r} dr = -2\pi k_i \int_{r_i}^{R_i} \frac{\partial \psi}{\partial r} dr. \qquad \text{(S2)}$$

Mass balance requires q to be constant. Eq. (S2) can thus be rewritten as

$$q_i = \frac{2\pi k_i (\psi_1 - \psi_2)}{\ln(R_i / r_i)}, \quad Q_i = \frac{2\pi k_i L_i (\psi_1 - \psi_2)}{\ln(R_i / r_i)}, \qquad \text{(S3)}$$

where ψ_2 is the water potential at the root-soil interface, ψ_1 is the average water potential of all xylem vessels in the stele. Eq.(S3) reveals that water uptake of a root segment does not depend on root diameter itself but on the ratio of root diameter to stele diameter. We call k_i in the above equations as intrinsic root water permeability to distinguish it from root hydraulic conductivity and root hydraulic conductance commonly used in the literature to describe the ability of roots to take up water. Radial root water permeability is the intrinsic ability of the peripheral cell layers to transport water from soil-root surface to xylem vessels.

Radial root hydraulic conductivity (k_R) used in calculating water uptake of a root segment is defined as follows in the literature.

$$Q_i = A_i k_R (\psi_1 - \psi_2), \quad A_i = 2\pi R_i L_i \qquad \text{(S4)}$$

where A_i is the surface area of the root segment. Comparing Eq. (S3) with Eq. (S4) gives

$$k_R = \frac{k_i}{R_i \ln\left(R_i / r_i\right)} \qquad \text{(S5)}$$

Eq. (S5) indicates that the radial root hydraulic conductivity depends not only the ability of the peripheral cell layers to transport water but also on root diameter and the ratio of the root diameter to the stele diameter (Fig. N1).

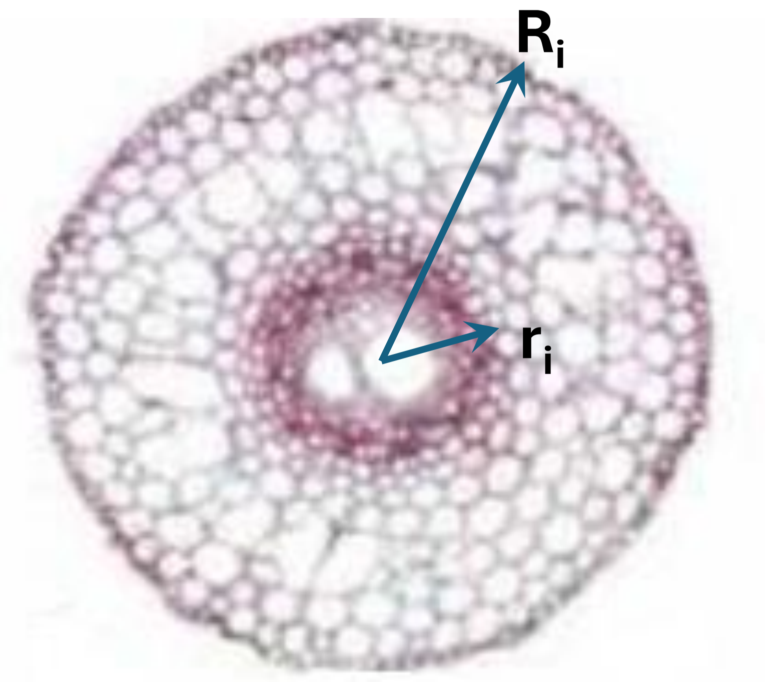

Fig. N1. Schematic show of water flow across the peripherical cell layers in a root segment

The radial hydraulic conductance (K_R) for calculating the ability of a root segment with length L_i and radius R_i to take up water is defined as follows in the literature:

$$Q_i = L_i K_R \left(\psi_2 - \psi_1\right) \qquad \text{(S6)}$$

Comparing Eq.(S6) and (S3) gives

$$K_R = \frac{2\pi k_i}{\ln\left(R_i / r_i\right)} \qquad \text{(S7)}$$

Eq. (S7) indicates that the radial hydraulic conductance depends not only on the ability of the peripheral cell layers to transport water but also on the ratio of root diameter to stele

diameters. Since $\ln(R_i / r_i) < 2\pi$, root hydraulic conductance is greater than radial root water permeability.

The units of radial water permeability, radial hydraulic conductivity and radial hydraulic conductance depend on the unit of water potential. When water potential is expressed as water head, the units of k_i, k_R and K_R are cm day^{-1}, day^{-1}, and cm day^{-1} , respectively. When water potential is expressed as pressure (MPa), the units of k_i, k_R and K_R are cm^2 day^{-1} MPa^{-1}, cm day^{-1} MPa^{-1} and cm day^{-1} MPa^{-1}, respectively, because the relationship between water head (H) and pressure (P) is $P = \rho g H$, where ϱ is the density of liquid water and g is the gravitational acceleration. We use water permeability in this paper because it better describes the response of the transport ability of the peripheral cell layers to environmental changes.
